# Silica Nanoparticles—A Versatile Tool for the Treatment of Bacterial Infections

**DOI:** 10.3389/fchem.2020.00602

**Published:** 2020-07-15

**Authors:** Vanitha Selvarajan, Sybil Obuobi, Pui Lai Rachel Ee

**Affiliations:** ^1^Department of Pharmacy, National University of Singapore, Singapore, Singapore; ^2^Drug Transport and Delivery Research Group, Department of Pharmacy, UIT The Arctic University of Norway, Tromsø, Norway; ^3^NUS Graduate School for Integrative Sciences and Engineering, Singapore, Singapore

**Keywords:** silica nanoparticles, mesoporous silica nanoparticles, antimicrobial, infectious diseases, antibiotic resistance, targeted delivery

## Abstract

The rapid emergence of drug resistance continues to outpace the development of new antibiotics in the treatment of infectious diseases. Conventional therapy is currently limited by drug access issues such as low intracellular drug accumulations, drug efflux by efflux pumps and/or enzymatic degradation. To improve access, targeted delivery using nanocarriers could provide the quantum leap in intracellular drug transport and retention. Silica nanoparticles (SiNPs) with crucial advantages such as large surface area, ease-of-functionalization, and biocompatibility, are one of the most commonly used nanoparticles in drug delivery applications. A porous variant, called the mesoporous silica nanoparticles (MSN), also confers additional amenities such as tunable pore size and volume, leading to high drug loading capacity. In the context of bacterial infections, SiNPs and its variants can act as a powerful tool for the targeted delivery of antimicrobials, potentially reducing the impact of high drug dosage and its side effects. In this review, we will provide an overview of SiNPs synthesis, its structural proficiency which is critical in loading and conjugation of antimicrobials and its role in different antimicrobial applications with emphasis on intracellular drug targeting in anti-tuberculosis therapy, nitric oxide delivery, and metal nanocomposites. The role of SiNPs in antibiofilm coatings will also be covered in the context of nosocomial infections and surgical implants.

## Introduction

Dubbed as a global epidemic, the emergence of antimicrobial resistance (AMR) has resulted in a dramatic increase in bacterial pathogens with resistance against one or multiple antibiotics (Dabke and Sheridan, 2011; Ferri et al., 2017; Merlino, 2017). The rise and spread of resistant pathogens mainly stem from frequent misuse of antibiotics, subsequent selection pressure and acquisition of genetic mutations that carry the resistant genes. At present, more than 2.8 million people in the United States are affected by antibiotic-resistant infections each year (Centers for Disease Control Prevention, 2019), with over 35,000 deaths recorded yearly for infections that were easily treatable in the past. It is estimated that by the year 2050, AMR could result in 10 million deaths per year with an estimated expenditure of $100 trillion (O'Neill, 2016). Methicillin-resistant *Staphylococcus aureus* (MRSA), drug-resistant *Clostridium difficile*, carbapenem-resistant *Enterobacteriaceae* (CRE), multidrug-resistant *Acinetobacter* are some of the high priority pathogens that require immediate attention and an action plan (World Health Organization, 2015). Moreover, some of these AMR strains are present in complex biofilm forms, raising an additional challenge for the treatment of chronic infections. This rising trend of AMR crisis is also accompanied by the absence of new antibiotic classes in the drug synthesis pipeline owing to impeding regulatory setbacks (World Health Organization, 2017a). The current set of antibiotics used for infectious disease treatment and even the last resort treatment drugs are all derived from the antibiotic classes discovered until 1984 (Gupta and Nayak, 2014).

Toward improving the efficacy of the available antibiotics, clinicians may resort to high drug dosing or an increment in dosage frequency. This recourse not only aggravates the existing toxicity and side effects of the antibiotics but also drives the development and spread of bacterial resistance. While research efforts on alternative antimicrobials such as antimicrobial peptides (AMPs) (Kang et al., 2014; Khara et al., 2014, 2015, 2016, 2017; Khara and Ee, 2015) and revisiting old forgotten antibiotics like octapeptins and actinorhodins (Theuretzbacher et al., 2015) are ongoing, it is necessary to identify novel strategies that render resistant pathogens vulnerable to existing antibiotics. The use of nanoparticles as a delivery vehicle for antimicrobials is one such strategy that could potentially combat the setbacks mentioned above. The benefits of nanomaterials in this application are manifold. By using nanoparticles as carriers, the mode of uptake by the pathogens can be tailored and thus circumventing issues associated with antimicrobial resistance mechanism such as hyperactive efflux pumps (Hadinoto and Cheow, 2014; Baptista et al., 2018; Vallet-Reg et al., 2019). In addition, nanoparticles can improve the pharmacokinetic profile of the drug by capitalizing on additional amenities such as optimal-drug loading and targeted delivery. Incidentally, this can also reduce the high drug dose generally administered to reach clinical efficacy reducing associated toxic side effects. Amongst a wide range of nanoparticles, silica nanoparticles (SiNPs) represent a unique class of inorganic nanoparticles with a wide array of functional features advantageous for combating bacterial infections (Karaman et al., 2018; Martínez-Carmona et al., 2018; Bernardos et al., 2019). There are many different types of SiNPs, such as the conventional non-porous SiNPs, mesoporous silica nanoparticles (MSN), hollow mesoporous silica nanoparticles (HMSN) and core-shell silica, either with or without surface modification. Particularly, MSN is a popular choice for targeted drug delivery given its flexible and desirable properties such as high drug loading capacity, tunable pore size and volume, ease-of-functionalization, and biocompatibility.

## Silica Nanoparticles: Synthesis and Properties

### Synthesis of Silica Nanoparticles

**Figure 1:**
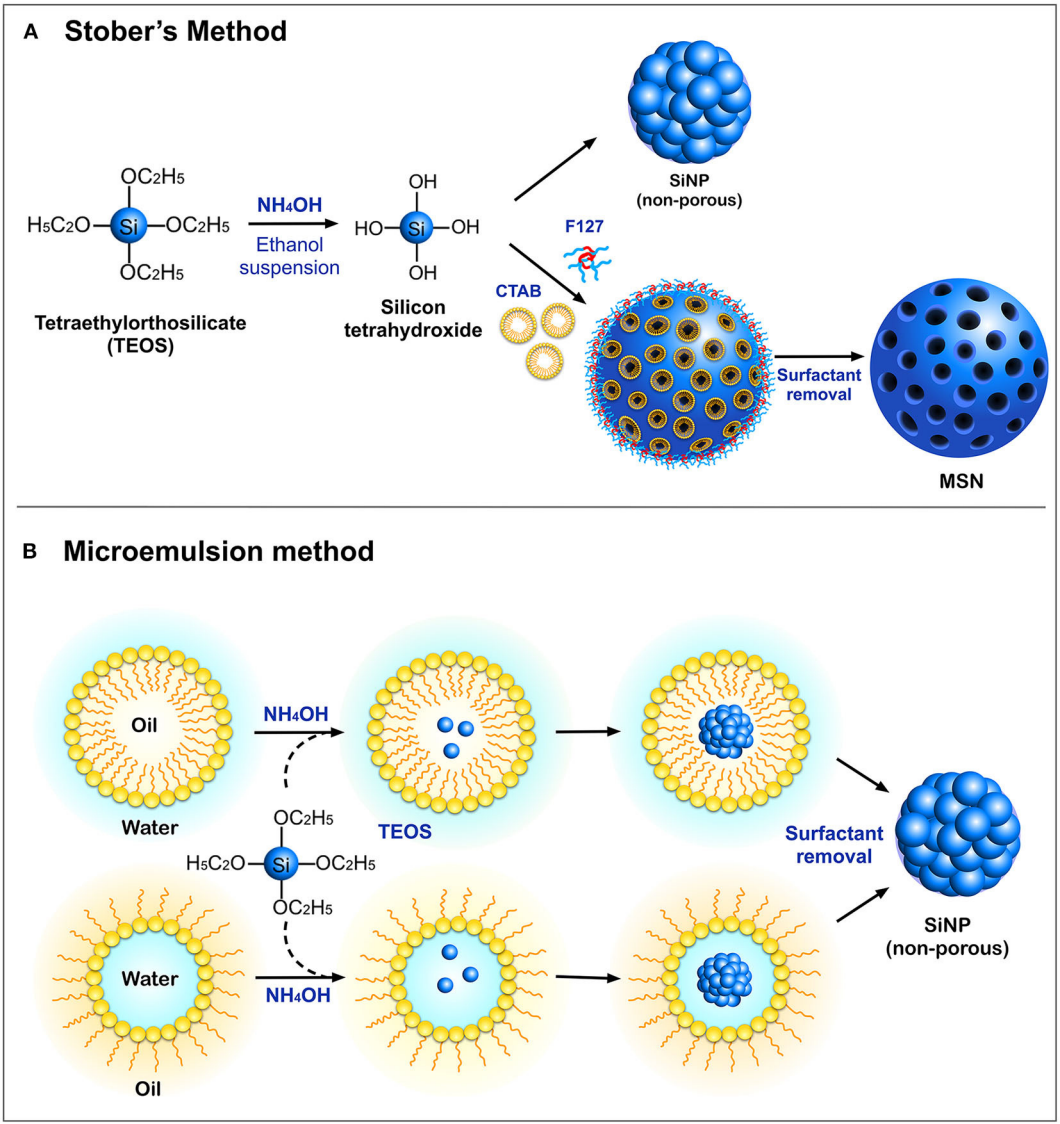
Schematic representation of different modes of synthesis of SiNPs **(A)** Stober's method and **(B)** Microemulsion method.

Silica nanoparticles can be synthesized by a number of protocols, yielding nanoparticles over a size range of 10–500 nm with a variety of shapes and physicochemical properties. The most commonly employed methods for the synthesis of SiNPs are the Stober's process and the microemulsion method (Figure 1). The Stober's method was first introduced in 1968, for the synthesis of monodispersed silica particles in the sub-micrometer range (Stöber et al., 1968). This technique utilizes a silica precursor, tetraethyl orthosilicate (TEOS) which in the presence of ethanol and ammonium hydroxide (NH_2_OH), undergoes hydrolysis followed by a polycondensation reaction to produce non-porous silica particles with sizes less than 200 nm. This synthesis protocol has now been fine-tuned to suit user-specific requirements (Rao et al., 2005). In addition to TEOS, other low-cost precursors such as sodium silicate solution (SSS) have been used (Zulfiqar et al., 2016a,b). The surface of these nanoparticles is rich in silanol groups which can be used as an anchor for surface modifications with organosilane coupling agents such as (3-aminopropyl) triethoxysilane (APTES) and (3-mercaptopropyl) trimethoxysilane (MPTMS) (Sterman and Marsden, 1966), to enable loading and adsorption of biomolecules. A modified Stober's process with the incorporation of surfactants such as cetyltrimethylammonium bromide (CTAB) and, site-directing agents such as the triblock copolymer (F127) is widely used to synthesize MSN with pore sizes ranging between 2 and 50 nm (Wu et al., 2013). These porous compartments are widely utilized for loading different drugs and biomolecules such as proteins, peptides, and DNA for various therapeutic and biomedical applications.

**Figure 2:**
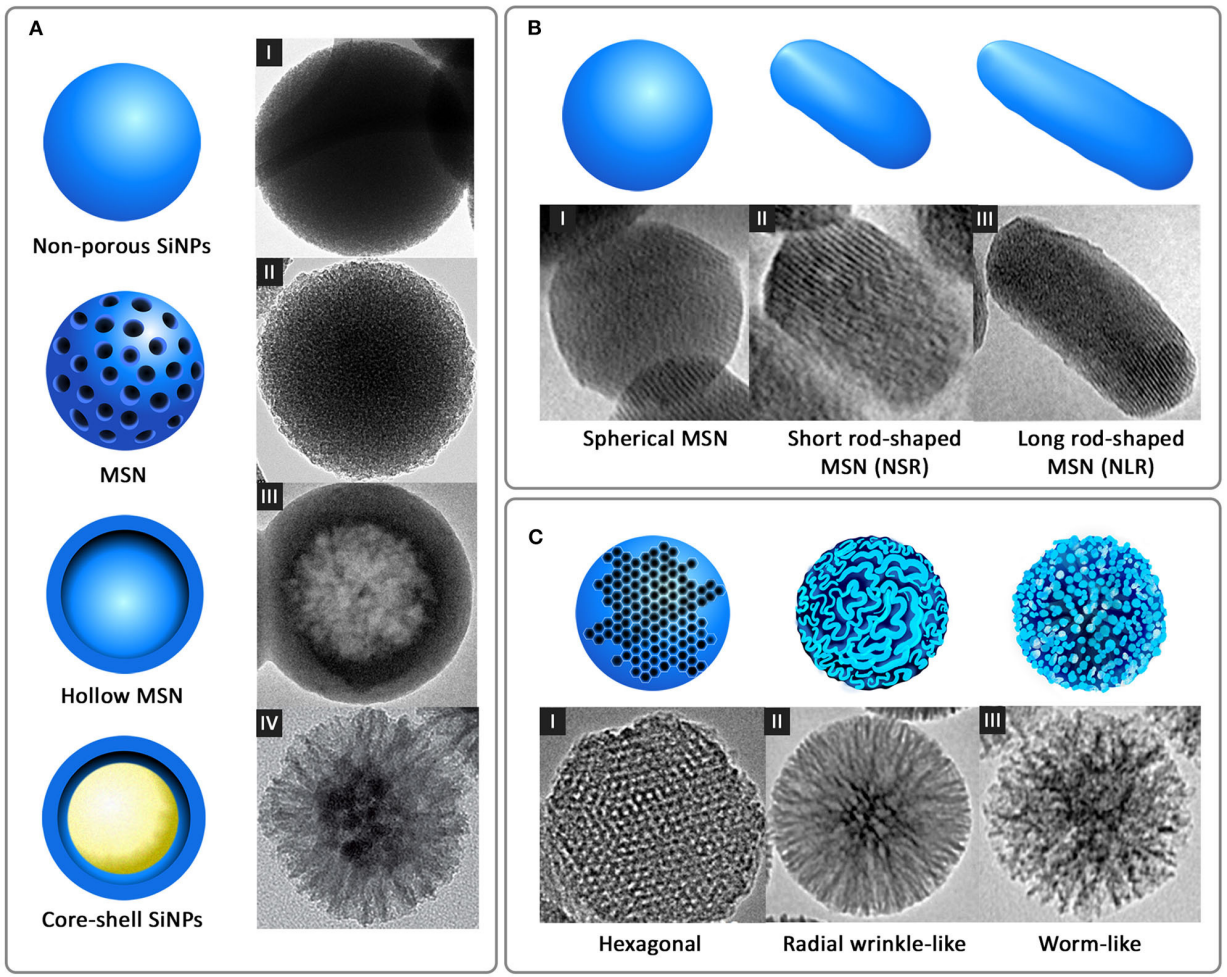
Different forms of silica nanoparticles based on **(A)** Structural morphology: I-non-porous SiNPs, II-MSN, III-hollow MSN [reprinted with permission from Springer Nature Limited 2017© (Bao et al., 2017)], and IV-core-shell SiNPs [reprinted with permission from WILEY-VCH Verlag GmbH & Co. KGaA, Weinheim 2016 © (Xiong et al., 2016)] **(B)** Shape: I-spherical MSN, II-short rod-shaped MSN (NSR), and III-long rod-shaped MSN (NLR) [reprinted with permission from Springer Nature Limited 2017 © (Zhao et al., 2017)] and **(C)** Pore morphology: I-ordered hexagonal [reproduced with permission from SAGE Publications Ltd 2014 © (Huang et al., 2014)], II-radial wrinkle-like [reprinted with permission from American Chemical Society 2012 © (Moon and Lee, 2012)], and III-worm-like [reproduced with permission from American Chemical Society 2013 © (Zhang et al., 2013)].

Another standard method for the synthesis of SiNPs is the microemulsion technique, which involves the formation of oil-in-water (O/W) micelles or water-in-oil (W/O) reverse micelles (Arriagada and Osseo-Asare, 1995; López-Quintela, 2003). These micelles stabilized by surfactants such as tweens or pluronics, act as nanoreactors for particle synthesis, and therefore, the size of the nanoparticles primarily depends on the volume of these nanoreactors. It is inside these nanoreactors that silica precursors undergo hydrolysis and condensation reactions to form SiNPs. It is also possible to load fluorophores and drugs into these nanoreactors to facilitate drug delivery applications. Other alternate methods such as low-temperature vapor-phase hydrolysis (Yan et al., 2014), spray drying (Cho, 2016), and chemical precipitation (Cai et al., 2009) have also been employed for the synthesis of SiNPs. The most common morphologies of SiNPs synthesized using the above methods are non-porous SiNPs, mesoporous silica nanoparticles (MSN), hollow mesoporous silica nanoparticles (HMSN) and core-shell SiNPs (Figure 2A).

### Structural and Functional Properties of SiNPs

#### Size, Shape, and Porosity

The physicochemical parameters of the nanoparticles such as size, shape, and porosity play a critical role in the delivery of payloads to the target site and its subsequent elimination from the body. Notably, size is an essential factor that governs the cellular uptake of the nanoparticles and its biocompatibility. In general, SiNPs are synthesized in size range between 10 and 500 nm. The particle size is generally controlled by varying the reaction parameters such as ammonia/sodium hydroxide concentration, mixing speed or the rate of TEOS addition. A functional, PEG-coated near-infrared (NIR) fluorescent silica nanoparticles called Cornell dots (C-dots) were designed and synthesized by Wiesner and team for molecular cancer imaging (Ow et al., 2005). The size of this FDA investigational new drug (IND)-approved silica nanoparticles was controllable even within 10 nm, in diameter.

Nanospheres and nanorods with different aspect ratios are the most common shapes of SiNPs (Figure 2B), synthesized for a wide variety of therapeutic and diagnostic applications. Many studies have shown that, like size, the shape also acts as a key player in modulating the physiological behavior and activity of nanoparticles. This difference in the physiological behavior was observed by Zhao et al. in a study wherein three different shapes of MSN, long rod nanoparticles (NLR), short rod nanoparticles (NSR) and spherical nanoparticles (NS) were analyzed for their *in vivo* oral bioavailability (Zhao et al., 2017). It was observed that the NLR displayed longer *in vivo* residence time, slower renal clearance and more prolonged blood circulation when compared to NSR and NS. In line with this, *in vitro* degradation experiments showed that NSR degraded at a faster rate compared to NS and NLR, attributable to the high specific surface area of NSR. In another similar study where the shape of fluorescent MSN was manipulated by varying the concentration of reagents, short rod MSN (NSR) and long rod MSN (NLR) with or without PEGylation were analyzed for biodistribution, clearance, and biocompatibility (Huang et al., 2011). It was shown again that NLR had longer blood circulation time, whereas NSR had a faster clearance rate from the body.

While size and shape are the primary determinants of uptake and biodistribution of nanoparticles, porosity played a stronger role in terms of payload delivery. Generally, the porosity of SiNPs can be controlled between 2 and 50 nm by varying synthesis parameters. This porous structure can be highly ordered as in the case of MCM-41-type silica nanoparticles with hexagonal pores or sometimes wrinkled or worm-like appearance (Figure 2C). Optimizing the pore size according to the size of the cargo and limit of controlled release is highly essential. While a smaller pore size might result in the restricted loading and release of the payloads at the target site, larger pore size can result in a premature release of the payloads before reaching the target site, causing unwanted side effects or toxicity. An interesting strategy to avoid premature release of payloads is the use of capping agents that enable drug release on specific triggers. Several capping agents for MSN such as cyclodextrin (Liu et al., 2016), polymers (Zou et al., 2013; Zhao et al., 2015), dendrimers (Nadrah et al., 2013; González et al., 2018), peptides/proteins (Climent et al., 2012; Braun et al., 2016; Chen et al., 2017; Cheng et al., 2017) have been reported. The effect of pore sizes of doxorubicin-loaded MSN on anticancer efficacies was studied by Li et al. (2018). Three variations of pore sizes, 2.3, 5.4, and 8.2 nm, indicated as MSN2, MSN5, and MSN8, respectively, were synthesized using microemulsion method. The results indicated that MSN2 exhibited the lowest loading capacity (8.2%) and MSN5 exhibited the strongest release profile and cellular uptake. The study highlights the importance of pore size control for better efficacy of the nanocarrier.

#### Surface Modification

**Figure 3:**
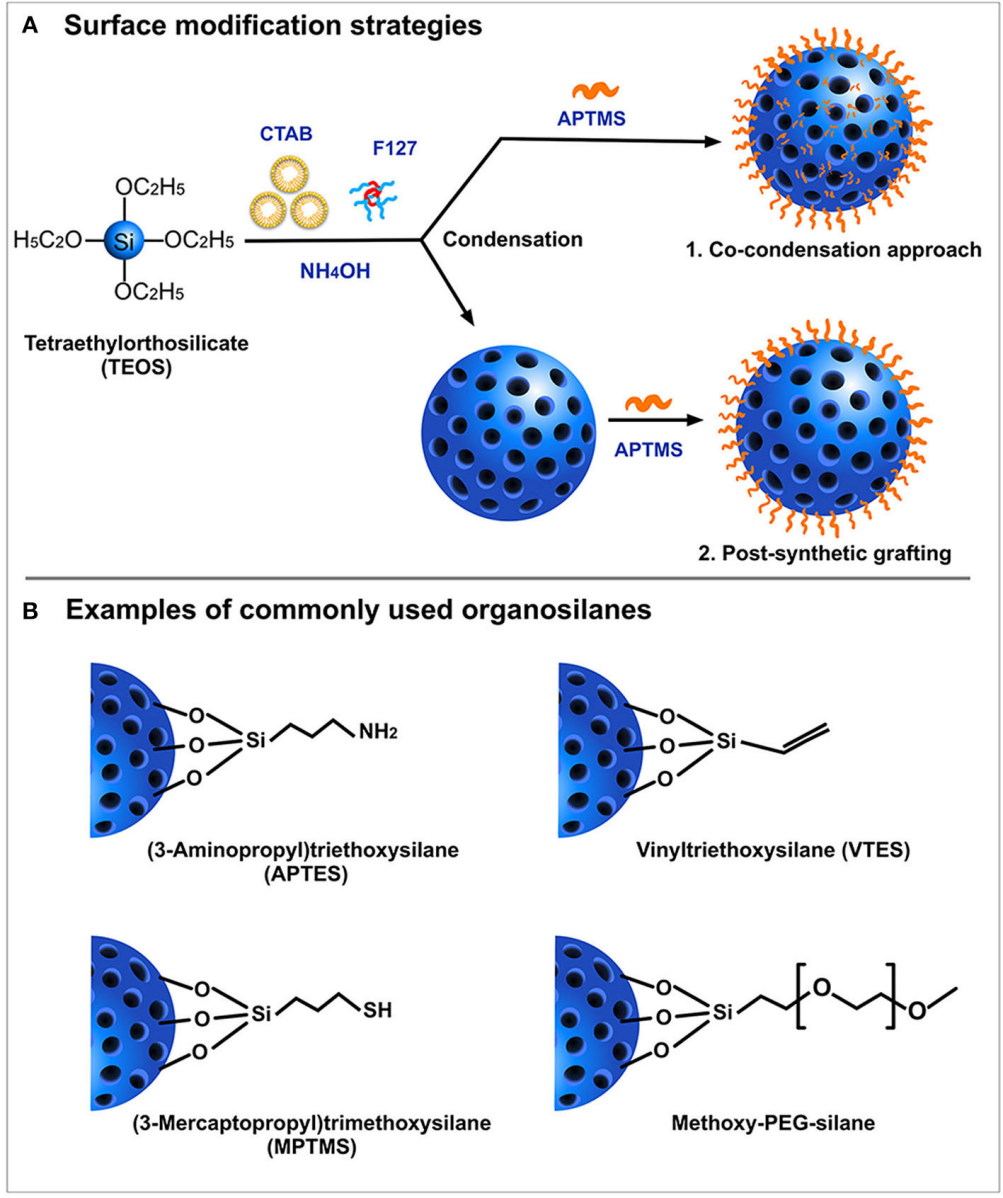
Schematic representation of **(A)** Surface modification strategies and **(B)** Examples of most commonly used organosilanes.

SiNPs are relatively easy to functionalize. Typically, the surface of SiNPs possesses a high content of silanol groups (Si-OH) which can be easily manipulated as the site of attachment for surface probes. This covalent modification strategy involves either co-condensation or post-synthetic grafting (Figure 3A) of different functional silanes (Figure 3B) onto the surface silanol groups. The post-synthetic grafting involves conjugation of functional groups, mostly on the surface of the nanoparticle, whereas the co-condensation approach entails the presence of modified functional groups even inside the pores of the nanoparticles. One primary reason to incorporate surface modifications is to improve the colloidal stability of the nanoparticles, which otherwise possess a high tendency to aggregate with each other. Polyethylene glycol (PEG) is often used as modifying agent as it can improve colloidal stability of SiNPs, and provide improved blood circulation time and enhanced biocompatibility (Jokerst et al., 2011; D'souza and Shegokar, 2016). Several studies have shown that PEG, being a hydrophilic polymer, forms a protective layer around the nanoparticles, effectively hiding the reactive surface groups. With a “stealth” mode of action, this modification prevents the binding of non-specific serum proteins, thereby avoiding early clearance from the circulation. In many cases, PEG also acts as a gatekeeper for the cargos loaded inside the pores of MSN (Cui et al., 2012; Jiao et al., 2016). Surface modifications also enable covalent conjugation of fluorescent probes (Sahoo et al., 2013), peptides (Luo et al., 2014; Shi et al., 2017; Sun et al., 2017), or drugs (Makarovsky et al., 2011; Qi et al., 2013) via several commercially available crosslinking agents such as N-(α-maleimidoacetoxy)succinimide ester (AMAS), *m*-maleimido-benzoyl-*N*-hydroxysuccinimide ester (MBS), and maleimide-PEG-*N*-hydroxysuccinimide (Mal-PEG-NHS) ester.

#### Biocompatibility and Biodistribution

While silica nanoparticles are generally considered non-toxic, specific properties of SiNPs, such as the size, reactive surface groups or sometimes the route of administration, can illustrate varied levels of toxicity in the body. Generally, acute systemic toxicity by SiNPs in blood cells is triggered by proinflammatory responses, oxidative stress, or the activation of the apoptosis pathway in addition to hemolysis of the red blood cells. Zhao et al. studied the effect of particle size on toxicity by examining the interaction of SBA-15 type and MCM-41 type MSN of sizes, 531 and 122 nm. respectively, on red blood cells (Zhao et al., 2011). Results showed that larger SBA-15 type MSN induced greater membrane distortion due to stronger adsorption and internalization, which resulted in subsequent hemolysis. On the contrary, smaller MCM-41 type MSN were adsorbed onto the red blood cells without inflicting deformity. In another study, FITC-tagged fluorescent SiNPs of variable sizes (850, 500, 250, and 150 nm) were assessed against RAW 264.7 macrophages cell lines wherein toxicological responses such as TNF-α production, LDH release, H_2_O_2_ release and ROS generation demonstrated size-dependent compatibility (Leclerc et al., 2012). In this study, the particles of size 850 nm showed a significant increase in the TNF-α and LDH release when compared to the control. However, the authors here used a fixed number of particles (1,000 particles/cell), irrelevant to overall size, volume or the surface area of the particles under study which could also be a contributing factor to the varied toxicological responses. These studies highlight the vitality of managing the physicochemical properties of SiNPs to ensure its safety. While studies on acute toxicity associated with SiNPs have been widely reported in the literature, a few studies have also investigated its chronic or sub-chronic toxicities. A study by Liu et al., demonstrated that mesoporous hollow SiNPs of size 110 nm showed a low toxicity in both single dose and sub-chronic exposure to mice (Liu et al., 2011). Here, in a single dose setting, LD50 was higher than 1,000 mg/kg and in a sub-chronic setting, exposure up to 80 mg/kg for 14 days did not result in mice death, thus indicating its low toxicity. Another elaborate study with a longer acute (10 days) and sub-chronic (60 and 180 days) exposure period studied a size and porosity dependent toxicity of SiNPs in both male and female mice (Mohammadpour et al., 2019). The results indicated that while smaller non-porous SiNPs (50 nm) and larger mesoporous SiNPs (500 nm) displayed more acute toxicity than larger non-porous SiNPs (500 nm), their sub-chronic toxicity was lesser compared to larger non-porous SiNPs (500 nm) after 60 days and 180 days post-intravenous injection.

Some studies have also investigated the role of administration route on the toxicity, biodistribution and elimination. A systematic evaluation of fate of SiNPs (110 nm) in the body upon intravenous, hypodermic, intramuscular and oral administration was conducted by Fu et al. (2013). While no histopathological abnormalities in target organs were detected with all four routes of administration, a slight inflammation was observed at the injection sites of hypodermal and intramuscular administrations. They also reported the persistent accumulation of the nanoparticles in liver in all routes of administration with hypodermic and intramuscular routes absorbed at a much slower rate compared to the oral and intravenous route. In another study, *in vivo* safety profile of SiNPs (100 nm) was compared between oral (low-dose-100 mg/kg; high-dose-1,000 mg/kg) and ocular topical delivery (10 mg/ml, drops, 4 times per day) (Kim et al., 2017). The toxicity profiles of different treatments for a period of 1 month revealed no significant damage to organs or cellular structures. A study on dermal silica toxicity which involved the application of colloidal SiNPs (20 nm) on the skin for 90 days, also did not show any significant toxicity or internal organ damage up to a dose of 2,000 mg/kg in rats (Ryu et al., 2014). Overall, fine tuning of physicochemical parameters such as surface charges and particle size can attenuate the toxicity and enhance *in vivo* biocompatibility of SiNPs.

In addition to biocompatibility, it is also essential to understand the elimination and degradation process of the nanoparticles while evaluating its safety profile. Studies have shown that the elimination of silica nanoparticles occurs either through feces or urine. Preferential elimination of SiNPs through either of these routes are dependent on size, surface area, or charge. Particles with larger size and surface area tend to eliminate though gastrointestinal tract via liver accumulation while smaller particles (less than 6 nm) preferentially eliminate through urinary tract. Some studies have also indicated a charge-driven excretion pattern in SiNPs. A study by Dogra et al. with SPECT/CT imaging and mathematic modeling approach reported that particles of size between ~32–142 nm displayed lower bioavailability with preferential accumulation in liver and spleen which can lead to hepatobiliary excretion via gastrointestinal tract (Dogra et al., 2018). However, particles with size less than 6 nm could rapidly undergo renal clearance which is considered effective in case of SiNPs developed for diagnostic applications like C-dots. The study also identified that cationic SiNPs tend to rapidly sequester through liver into gastrointestinal tract compared to its anionic counterpart, similar to the findings from another charge-mediated excretion study of SiNPs (Souris et al., 2010). Also, few studies have also explored the fate of SiNPs targeted for intracellular delivery. On this account, a study by Shi et al. explored the intracellular and extracellular degradation of different PEGylated SiNPs (biocomposite, multilayered, and hollow mesoporous) and its dissolution kinetics in buffer, culture medium and in the presence of human dermal fibroblasts (NHDF) (Shi et al., 2018). Their results on degradation pathways of SiNPs with different internal structures postulates a hydrolytic degradation process rather than a biodegradation pathway. However, there was no strong correlation between the internal structures of the nanoparticles and rate of dissolution. A recent study by Moller and Bein, reported the influence of synthesis pH, composition, surface modification and size on the aqueous degradation rates of SiNPs (Möller and Bein, 2019). The results indicated that particles with co-condensed functional groups and those synthesized in basic condition degraded at a much faster rate than purely siliceous MSN. Additionally, when co-condensed particles were modified with disulfide bridged silanes on the surface, degradation rate was slower. These studies clearly indicate that degradability and elimination of SiNPs can be carefully tweaked by varying the size, structure, and its functional properties.

## Applications of Silica Nanoparticles in Antimicrobial Therapeutics

Nanoparticle-based drug delivery has been established as one of the most promising therapeutic strategies owing to its versatility and enhanced functionality to overcome physiological barriers. In this regard, silica nanoparticles (SiNPs) have been proven to be a lucrative choice for many biomedical applications, especially cancer and antimicrobial therapeutics. The versatility of SiNPs is particularly advantageous for antimicrobial therapeutics, including biofilm treatment, given the rising challenge of antimicrobial resistance. Since these nanoparticles can attack pathogens by multiple modes including physical damage to cell membranes, ROS production and endo-lysosomal burden, in addition to the antimicrobial activity induced by the cargo itself, the window for the development of antimicrobial resistance is quite narrow. Thus, this section highlights different modes of drug payload delivery of SiNPs targeted against bacterial pathogens with emphasis on anti-tuberculosis (anti-TB) therapy which requires intracellular targeting. In addition, other delivery compositions of SiNPs in infectious disease treatment such as metal-silica nanocomposites, nitric oxide (NO) delivery, antibiofilm coatings and dental composites were also explored and are summarized in Supplementary Table 1.

### Targeted Delivery of Antimicrobials

Silica nanoparticles, mainly MSN, have been widely exploited for their application in the delivery of drugs and other biomolecules such as proteins, peptides, and nucleic acids. Although the use of antibiotics is the conventional treatment modality for infectious diseases, requirements for high drug dosages *in vivo* and its association with resistance is a primary concern. In addition to unwarranted toxicity associated with direct delivery of antibiotics, another popular class of antimicrobials called antimicrobial peptides (AMPs) (Kang et al., 2014) are additionally prone to proteolytic degradation at the infection sites heavily impairing its activity. This, however, can be evaded by the effective use of nanotechnology which offers lucrative advantages such as high loading capacity, site-directed delivery and in some cases, triggered drug release.

#### Drug Loading

**Figure 4:**
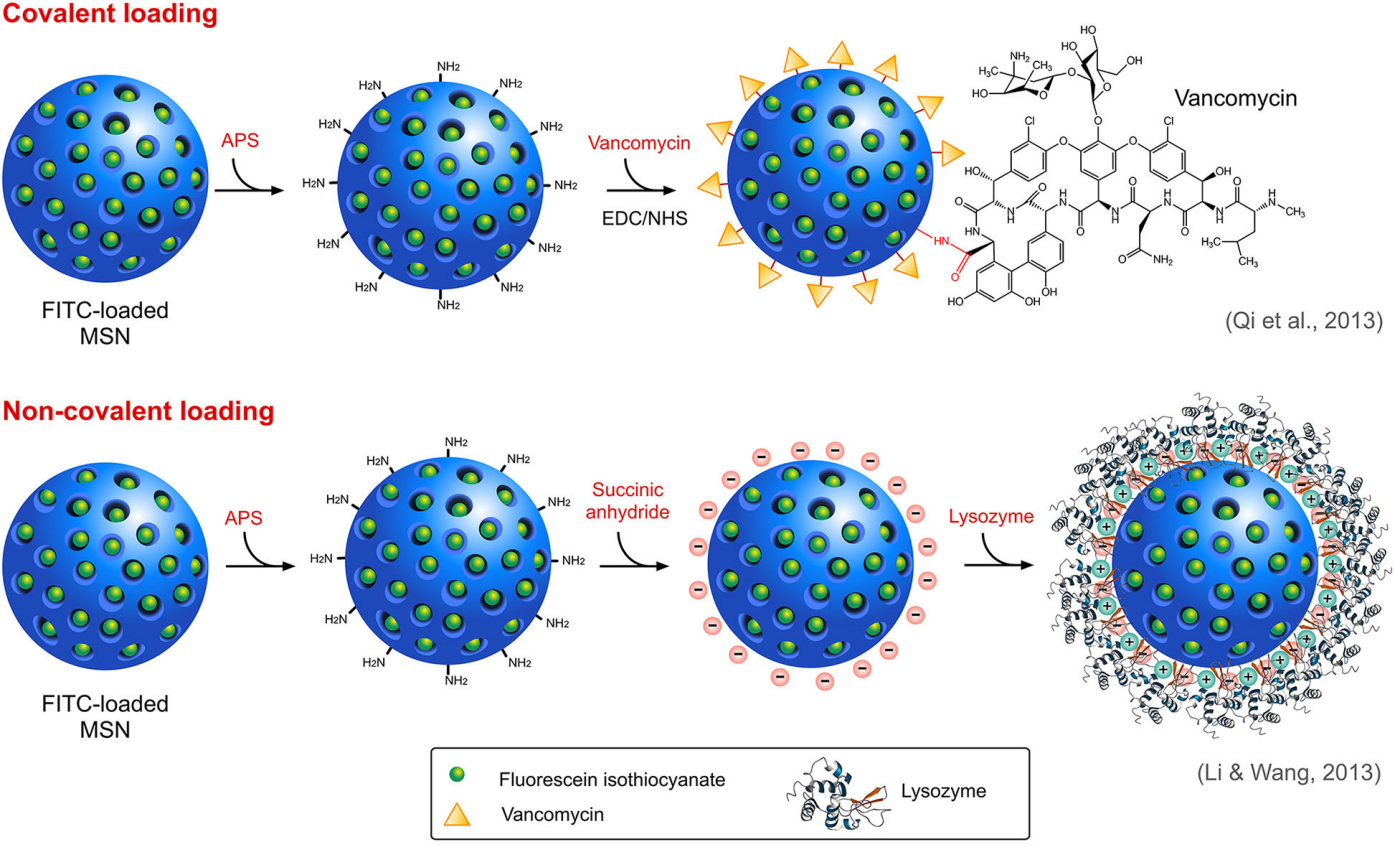
Different modes of loading of antimicrobials onto MSN.

A wide variety of antimicrobials such as antibiotics, peptides, and other functional materials can be loaded onto SiNPs, either covalently or non-covalently (Figure 4), offering a rational solution to the concerns associated with resistance, physiological barriers such as enzymes or serum proteins and other functional barriers such as drug solubility or toxicity. Covalent conjugation or grafting of the antimicrobials onto the surface of modified SiNPs involves the use of different linker molecules such as PEG crosslinkers and silane-coupling agents. For instance, an FDA approved antimicrobial triclosan (Irgasan) was covalently linked to the surface of SiNPs via a silane coupling agent, 3-(triethoxysilyl)propyl isocyanate (TESPC) (Makarovsky et al., 2011). Upon interaction with bacteria, certain membrane-associated enzymes ensue slow release of triclosan from SiNPs, which resulted in a superior bactericidal activity. In another similar study, vancomycin was directly conjugated to amine-modified, FITC-loaded MSN via EDC/NHS reaction for selective targeting and killing of Gram-positive bacteria (Qi et al., 2013). The same research group also studied the antibacterial activity of lysozyme, an antibacterial enzyme capable of inflicting membrane damage in bacteria, coated onto FITC-loaded MSN (Li and Wang, 2013), with the difference being the electrostatic interaction between the enzyme and the SiNPs, as opposed to the covalent interaction of vancomycin in the previous study. Non-covalent modes of interaction such as electrostatic attraction is employed predominantly in cases where the drug molecules are loaded either inside the pores of MSN or on the surface modified SiNPs. Like lysozyme, several antimicrobial peptides (AMPs), also known as host defense peptides have also been loaded onto SiNPs. AMPs, generally touted as an alternative to conventional antibiotics, are cationic and amphiphilic molecules known to induce damage in the bacterial membranes. Its inherent proteolytic instability necessitates the use of effective nanocarriers like SiNPs for delivery at the infection sites. Cationic nature of AMPs could enable its successful loading onto anionic, surface modified SiNPs via strong electrostatic interaction. On this account, Braun et al., reported that loading of a cationic AMP, LL-37 onto anionic calcined MSN (MSNc) (≈ −35 mV) was higher than its loading onto cationic aminated MSN (MSNa) and non-porous SiNPs (NSN) (Braun et al., 2016). Lower loading onto NSN even with high negative charge (≈ −55 mV) was attributed to its lower surface area. In this study, they also reported an interesting correlation between surface charge, loading and membrane interaction. The membrane interaction studies indicated that MSNc even with high loading of LL-37 did not interact with the negatively charged membrane whereas NSN with a low but strong loading of the peptide was able to interact with the membrane while exerting a slow release profile. In addition, MSNa due to its positive charge was able to strongly interact with the membrane even without peptide loading which also correlates with its high hemolytic activity. Similarly, Kwon and team used the electrostatic interaction between cationic peptide and phosphonated-porous silicon nanoparticles for the delivery of tandem peptide cargo made of lactoferrin and a synthetic bacterial toxin _D_[KLAKLAK]_2_ for treating *P. aeruginosa* infection in lungs (Kwon et al., 2017). An interesting study by Behzadi and colleagues involved loading of nisin, a natural food preservative, covalently and non-covalently onto MSN with different surface chemistries such as amination, carboxylation and polyethyleneimine (PEI)-coating with intent to impart stability and enable sustained release and activity (Behzadi et al., 2018). It was found that the mode of interaction indeed affected the antimicrobial activity profile of the nisin-loaded MSN. Irrespective of the surface chemistry, covalent conjugation of nisin resulted in much lower activity compared to those under electrostatic attraction which can be attributed to lower bioavailability due to stable conjugation. The results of the study highlight the importance of choosing the right mode of interaction for the right type of application.

In addition to the mode of interaction, drug loading can also be achieved in two ways, either during synthesis or post-synthesis while post-synthesis drug loading results in encapsulation of drug onto the surface or inside of the pores, addition of a drug during synthesis results in the incorporation of the drug in the core framework of the nanoparticle itself. Stewart and coworkers demonstrated the co-assembly of an antimicrobial drug (Octenidine dihydrochloride, OCT) and silica with a loading efficacy of 35 wt% (Stewart et al., 2018). The study claims that with such a system, a lower drug release rate for a longer period can be achieved as opposed to the initial burst release of the conventional drug formulations. A similar method of drug encapsulation was achieved by Capeletti and team where they encapsulated tetracycline (TC) antibiotics into silica nanospheres during synthesis to prepare SiO_2_-TC nanocomposites (Capeletti et al., 2014). In this study, both bare SiO_2_ NPs and SiO_2_-TC nanocomposites exhibited superior antimicrobial activity against both susceptible and TC-resistant *E. coli* strains.

#### Drug Delivery

**Figure 5:**
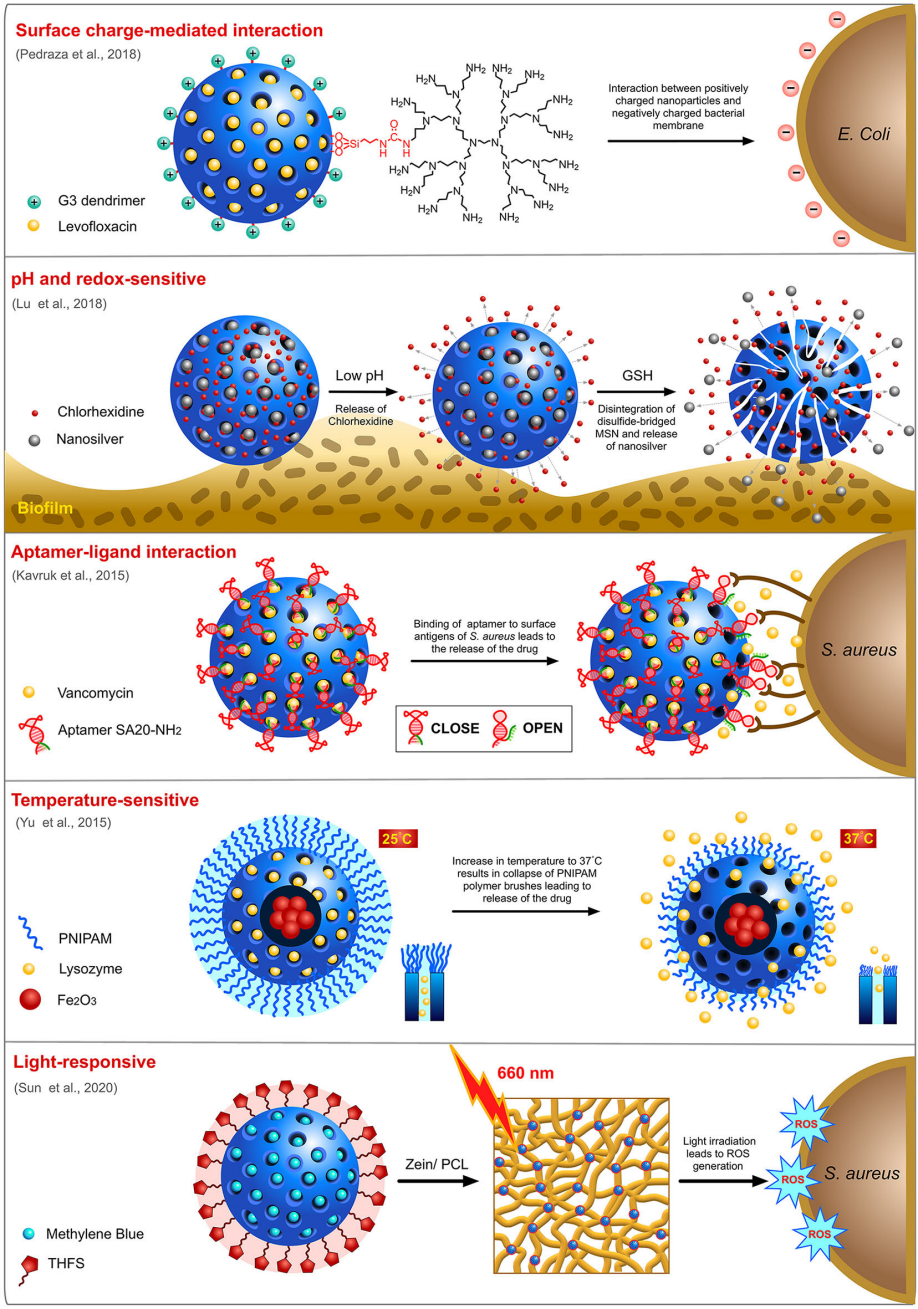
Targeted delivery of antimicrobials by MSN using different strategies.

Many different strategies have been employed for the delivery of targeted delivery of antimicrobials to bacteria (Figure 5). Charge of a nanocarrier can invariably affect its ability to interact with negatively charged bacterial membrane and influence its selectivity. Pedraza and team designed a “nanoantibiotic” system made of MSN loaded with levofloxacin (LEVO) and surface functionalized with positively-charged *N*-(2-aminoethyl)-3-aminopropyltrimethoxysilane (DAMO) which acts as a targeting agent rendering affinity toward negatively-charged bacterial membrane and biofilms (Pedraza et al., 2018). Antibiofilm activity of the this LEVO-loaded nanocarrier against *S. aureus* indicated a complete destruction biofilm post-treatment. The same team previously reported a LEVO-loaded MSN grafted with poly(propyleneimine) dendrimer of third generation (G3) that can effectively penetrate cellular membrane of *E. coli*, a Gram-negative bacteria with excellent antibiofilm activity (González et al., 2018). This penetration efficacy was ascribed to the presence of positively charged G3 dendrimers on the surface of MSN which can interact with the negatively charged bacterial cell membrane.

Despite being one of the most promising nanocarriers, MSN still suffers from issues associated with the premature release of cargo, which results in unwanted drug side effects and low bioavailability at the site of infection. In order to achieve a well-controlled release of cargos, only at the site of infection, designing a “smart” delivery vehicle that can sense the stimuli from the environment to open the pores is essential. Stimuli-responsive drug release has been widely exploited for cancer therapeutics wherein acidic pH, and the difference in redox potential at the cancer microenvironment are used as common triggers for the drug release. Likewise, for a bacterial infection site, drop in pH during active anaerobic metabolism can be utilized as a trigger to enable the spatiotemporal release of encapsulated drugs. Utilizing this knowledge, Kuthati and team designed a pH-triggered MSN nanocarrier wherein silver-indole-3 acetic acid hydrazide (IAAH-Ag) complex was linked to IBN-4 type MSN via weak pH-sensitive hydrazone bonds (Kuthati et al., 2015). As hypothesized, the release profile of silver ions from the transitional metal complexes at acidic pH 5.0 was much higher (70%) than the physiological pH 7.4 (25%). Chen and team designed a pH-responsive MSN (MSN@FA@CaP@FA) with double folic acid (FA) and calcium phosphate (CaP) electrostatically coated on the surface of the MSN (Chen X. et al., 2018). This nanocarrier loaded with ampicillin displayed potent antimicrobial activity against *E. coli* and *S. aureus*. In another study, Lu and team fabricated a biodegradable, silver-decorated, chlorhexidine (CHX)-loaded MSN (Ag-MSN@CHX) for oral biofilms with the acidic-redox environment (Lu et al., 2018). While the redox microenvironment of the biofilm dismembers the disulfide-bridged framework of MSN, consequently releasing the silver ions, the acidic pH helps in the dissociation of CHX from the carboxyl functional groups of MSN. It was observed that Ag-MSN@CHX was able to restrict the formation of *S. mutans* biofilm more efficiently than the individual components (AgNO_3_, Ag-MSN, and CHX). Li and his team also developed and extensively studied two pH-sensitive nanovalves on MSN surface for the delivery of a fluoroquinolone, moxifloxacin (MXF) and tested its efficacy against *Francisella tularensis-*infected macrophages (Li et al., 2015). The first nanovalve is composed of aniloalkane (ANA) stalk and α-cyclodextrin (α-CD) as capping molecule while the second nanovalve is composed of 1-methyl-1H-benzimidazole (MBI) stalk and β-cyclodextrin (β-CD) as the capping molecule. With protonation of the stalk at lower pH, nanocarrier systems, MSN-ANA-MXF and MSN-MBI-MXF dissociate the capping molecules triggering the release of MXF. Overall, MSN-MBI-MXF possessed better uptake and release capacity than MSN-ANA-MXF. An aptamer-gated vancomycin-loaded MSN was designed by Kavruk and team for site-directed controlled release of antibiotics (Kavruk et al., 2015). Here, the aptamers are specific to antigens present on the surface of *S. aureus* bacteria and thus a controlled release of vancomycin was achieved upon interaction of the aptamers with the bacterial surface. A temperature-sensitive nanocarrier release system was proposed by Yu et al. (2015). They fabricated a core-shell MSN system for loading of the antibacterial enzyme, lysozyme (Lys) with iron oxide (Fe_3_O_4_) core and a mesoporous silica shell which was further coated with poly(*N*-isopropylamide) (PNIPAM), a temperature-sensitive polymer known to collapse at higher temperatures. This carrier system with better and steady release of enzyme at 37°C was further tested for its antimicrobial activity against *Bacillus cereus* and *Micrococcus luteus*. It was observed that while no temperature-dependent activity was observed at a lower concentration (0.25 mg/ml), at a higher concentration (0.5 mg/ml), a temperature-dependent activity profile was observed with incubation at 37°C yielding more activity than 28°C.

Photodynamic therapy is another technology widely evaluated for strategic release of antimicrobials from the nanocarriers by application of external stimuli. A few recent studies have highlighted this potential in MSN nanocarriers using different photosensitizers (PS). A study by Paramanatham and team encapsulated malachite green, a cationic photosensitizing molecule into MSN and evaluated its antimicrobial potential against *E. coli* and *S. aureus* by laser irradiation at a wavelength of 670 nm (Paramanantham et al., 2019b). The results indicated that antibacterial efficacy effected by reactive oxygen species (ROS) generation of MG-MSN was higher in *S. aureus* than *E. coli* which could be due to the complexity of outer membrane of *E. coli*, a Gram-negative pathogen. The same team also evaluated the potential of toluidine blue (TB), another photosensitizer on MSN encapsulation and activity against biofilms formed by *P. aeruginosa* and *S. aureus* (Paramanantham et al., 2019a). The results indicated that TB-MSN was able to effectively inhibit biofilm formation in both bacterial species. In another study, methylene blue (MB)-loaded MSN embedded onto an electrospun matrix made of zein and polycaprolactone (PCL) (Sun et al., 2020) was studied for its antimicrobial efficacy. On laser irradiation at a wavelength of 660 nm, the nanocomposite membrane displayed a drastic reduction in the survival rate of both *E. coli* and *S. aureus* mediated by the release of ROS.

##### Intracellular targeting for anti-tuberculosis therapy

According to the World Health Organization (WHO), tuberculosis (TB) is considered one of the leading causes of morbidity and mortality in the world, encompassing around 1.6 million deaths in 2017 (World Health Organization, 2017b). While a wide array of effective antibiotics such as isoniazid (INH), rifampicin (RIF) and pyrazinamide (PZA) are available, these antibiotics possess poor pharmacokinetic properties which often causes toxic side effects. The rapid emergence of drug resistance to these first-line treatment drugs also resonates the requirement of a drug delivery system that can efficiently deliver high drug concentrations to target macrophages. MSN, with its high pore loading capacity and stability, could be a valuable choice for anti-tuberculosis therapy. On this cue, Clemens and colleagues developed surface-modified MSN-based delivery systems (Clemens et al., 2012). The authors investigated (i) unmodified MSNP; (ii) a cationic PEI functionalized MSNP; and (iii) MSNP equipped with a pH-dependent nanovalves made of β-cyclodextrin. The results indicated that both PEI-coated MSNP loaded with RIF and the pH-sensitive-MSNP loaded with INH displayed superior antimicrobial activity than the uncoated MSNP and free drugs. The same team also investigated a pH-responsive INH-loaded MSN where the drug was conjugated to PEI-PEG via hydrazone bond formation (Hwang et al., 2015). It was shown that the designed drug system was not only ingested by the *M. tuberculosis*-infected human macrophages effectively but also was able to kill the intracellular pathogens in a dose-dependent manner. In another study, the team attempted to load a water-insoluble anti-TB drug clofazimine (CFZ) into MSN using an additive chaperone, acetophenone (AP) instead of conventional loading methodology (Chen W. et al., 2018). This optimized protocol yielded them 4.5 times more loading and 2,300 times more release with efficient killing profile comparable to treatment with CFZ alone. Another team also sought the use of MSN for the delivery of PA-824, a poorly soluble antimicrobial agent and moxifloxacin (MFX) to target *M. tuberculosis*-infected macrophages (Xia et al., 2014). This system displayed a comparable antimicrobial activity to the pristine drugs. Previously, Zhu and team developed a dual-functional composite scaffold drug delivery system (CS-DDS) where drug-loaded MSNs were coated onto a β-tricalcium phosphate (β-TCP) scaffold with additional bioactive glass coating for osteoarticular tuberculosis therapy (Zhu et al., 2011). While the loaded anti-TB drugs, INH and RIF can contribute toward infection treatment, β-TCP scaffolds could aid in the recovery of residual cavity. In addition to antibiotics, many antimicrobial peptides have also shown efficient anti-TB activity in recent years (Khara et al., 2015, 2016). Like the common challenge with anti-TB drugs is their poor solubility, peptide drugs face challenges associated with proteolytic degradation. Hence, an efficient nanocarrier like MSN is vital to execute the delivery of peptides to the target site. In a recent study, Tenland and team loaded an effective anti-TB peptide, called NZX into mesoporous silica nanoparticles to improve its delivery efficacy (Tenland et al., 2019). *In vivo* studies suggested that NZX-loaded mesoporous silica nanoparticles were able to significantly reduce the bacterial load compared to the RIF treatment.

### Other Delivery Composites

#### Metal-Silica Nanocomposites

Some metals such as copper (Cu) and silver (Ag) with inherent antimicrobial activity have been used to evade infections since ancient times. However, they exhibit high cytotoxicity toward the mammalian cells and possess poor pharmacokinetic properties. Thus, incorporating these metals as co-delivery payloads with SiNPs has proven effective for antimicrobial applications. For instance, an MCM-41 type MSN with encapsulated silver nanocrystals (Ag@MESs) in a yolk/shell fashion was synthesized and tested against *B. anthracis* and *E. coli* (Liong et al., 2009). It was observed that Ag@MESs were able to inhibit bacterial growth in both the species. While the nanocomposite was able to slow the growth of *B. anthracis* at 20 μg/mL and completely inhibit growth at 100 μg/mL, it produced no such noticeable effect in *E. coli*. In another study, silver-containing silica nanorattles (Ag@SiO_2_) were synthesized and evaluated for their antimicrobial activity against *E. coli* and *S. aureus*. They were also tested for their cytotoxicity against immune cells. The results indicated a strong bactericidal activity with no immunotoxicity or proinflammatory response. Karaman and team investigated silver ion-doped MSN of three different sizes and shapes with outer chitosan coating [Cht/MSP (1/2/4): Ag^+^] for their antimicrobial potential against *E. coli, S. aureus*, and *V. cholerae* (Karaman et al., 2016). They also studied the combinatorial effect against *V. cholerae* by incorporating the antibiotic kanamycin. The results indicated a good antimicrobial activity against the three strains tested with no cytotoxicity at the tested concentrations. Incorporation of kanamycin as a combinatorial treatment with Cht/MSP (1/2/4): Ag^+^ resulted in better activity compared to when treated alone.

Like silver, copper is another common heavy metal capable of wielding bactericidal activity. Compositing Cu with SiNPs can help in sustained release of Cu to exert antibacterial activity. Synthesis of core-shell copper-silica nanoparticles for antimicrobial activity was reported by Maniprasad and Santra (2012). The results indicated that the core-shell nanoconstructs exhibited better antibacterial efficacy against *E. coli* and *B. subtilis* than the insoluble Cu-OH particles. Similarly, Kim et al. studied Cu deposition on the surface of spherical SiO_2_ nanoparticles synthesized with (Cu-SiO_2_-3/4) or without (Cu-SiO_2_-1/2) catalyst and investigated its antimicrobial property (Kim et al., 2006). The results clearly showed that Cu-SiO_2_-3 nanocomposites exerted a superior antimicrobial activity against fungi and both Gram-negative and Gram-positive bacteria.

#### Nitric Oxide (NO) Delivery

Nitric oxide (NO) is a free radical released by immune cells in response to infections. It also acts as a vasodilator and a tumoricidal agent. NO has been shown to possess broad-spectrum antimicrobial activity with the aid of small molecule NO donors such as sodium nitrite, diazeniumdiolates, and *S*-Nitrosothiols group (Schairer et al., 2012). However, spontaneous decomposition of these NO donors emphasizes the need for a delivery scaffold that prolongs the decomposition process and enables controlled release. In 2007, Shin et al. synthesized and characterized NO-releasing SiNPs using *N*-diazeniumdiolate NO donors with different aminoalkosilanes, (Aminoethylamino-methyl) phenethyltrimethoxysilane (AEMP3), *N*-(6-aminohexyl)aminopropyltrimethoxysilane (AHAP3), and *N*-(2-aminoethyl)-3-aminopropyltrimethoxysilane (AEAP3) (Jae et al., 2007). The same group also tested the anti-biofilm activity of these nanoparticles synthesized with either AHAP3 or *N*-methylaminopropyltrimethoxysilane (MAP3) against *P. aeruginosa, E.coli, S. aureus, S. epidermidis*, and *C. albicans* (Hetrick et al., 2009). Results indicate that MAP3 nanoparticles exerted a 1,000-fold more antibiofilm activity than AHAP3-nanoparticles. Additionally, MAP3-nanoparticles were also the most effective against the biofilm formed by Gram-negative bacteria (*E. coli* and *P. aeruginosa*), followed by *C. albicans* and were least effective against Gram-positive bacteria (*S. aureus* and *S. epidermidis*). In one study, Hetrick *et al*. attempted to validate the requirement of nanoparticle for NO delivery rather than just the donor NO, in this case, 1-(2-(carboxylate)pyrrolidin-1-yl)diazen-1-ium-1,2-diolate (PROLI/NO), by evaluating their antibacterial efficacy against *P. aeruginosa* (Hetrick et al., 2008). The results indicated that NO-releasing nanoparticles are more effective in killing *P. aeruginosa* than the small molecule donor, PROLI/NO. Notably, PROLI/NO incurred significant toxicity against mammalian fibroblasts while NO-releasing SiNPs showed no such toxicity.

#### Antibiofilm Coatings and Dental Composites

Microbial biofilm formation is recognized as a key virulence factor in localized chronic infections and a prominent menace in nosocomial infections (Koo et al., 2017). Within biofilms, microbes develop tolerance to conventional antibiotics, given the poor diffusion capacity of these agents. With only a few therapeutics currently in the pipeline, the control of biofilm formation and eradication of preformed biofilms is truly daunting. Incidentally, SiNPs have proven to be indispensable against biofilms with effective inhibitory action on wearable medical implants. Kanugala and colleagues studied the anti-biofilm potential of phenazine-1-carboxamide (PCN)-loaded MSN, coated on silicone urethral catheters (Kanugala et al., 2019). The antimicrobial activity of native MSNPs, PCN, and PCN-MSNPs was evaluated against planktonic *C. albicans, C. albicans* biofilm, and *C. albicans*-*S. aureus* polymicrobial biofilm. The results demonstrated a superior antimicrobial and antibiofilm activity of PCN-MSNPs compared to native MSNPs and PCN alone. Moreover, silicone urethral catheters coated with PCN-MSNPs did not exhibit any polymicrobial biofilm formations. Similarly, Wang et al. incorporated silica-gentamicin nanoparticles within a gelatin matrix and crosslinked on microarc-oxidized titanium as a coating for percutaneous implants (Wang et al., 2017). It was shown that the antibacterial titanium coating possessed good biocompatibility and was able to inhibit the growth of *S. aureus*. Co-deposition of PEI-functionalized SiNPs during electroless nickel plating, a robust hard coating technique employed for ferrous metal, was studied by Huang and team (Huang et al., 2016). They compared the antimicrobial and antibiofilm activity of this system along with a polytetrafluoroethylene (PFTE) NPs-coated system. While the native and Ni-PFTE-modified stainless steel did not exhibit antimicrobial activity, Ni-SiNP^+^-modified steel inactivated *L. monocytogenes* efficiently. However, Ni-SiNP^+^-modified steel exhibited a lower anti-biofilm activity compared to Ni-PFTE coating. A co-deposition of Ni-SiNP^+^ with PFTE nanoparticles offered both antimicrobial and anti-fouling nature to the coating.

In addition to the different metallic biomedical implants discussed above, drug loaded SiNPs can be used as an antimicrobial additive for bone cement and dental matrices. Poly (methyl methacrylate) (PMMA) is one of the most common commercially available bone cement matrices for dental and bone implants. Direct loading of antimicrobials onto this matrix ensue difficulties in achieving a sustained drug release due to poor mixing or uneven distribution. Thus, SiNPs can act as a reinforcement material toward achieving controlled drug release (Letchmanan et al., 2017). In one study, PMMA matrix incorporated with amphotericin B-loaded MSN (0.5, 1, 2.5, or 5 wt%) were evaluated for their anti-adherent effect against oral *C. albicans* and *S. oralis* and long-term anti-microbial efficacy against *C. albicans* (Lee et al., 2016). Amongst the variants, 2.5 and 5 wt% displayed better anti-adherent properties with increased surface roughness and energy. It was also observed that 2.5 wt% MSN-PMMA exhibited a long-term antimicrobial efficacy (17 days). The same group also investigated silver-sulfadiazine (AgSD)-loaded MSN in place of amphotericin B with the aim of conferring long-lasting microbial anti-adhesive effect (28 days) (Jo et al., 2017). The advantage of this drug system lies in the “rechargeable” nature of the drug matrix which resulted in a renewed long-term microbial anti-adhesive effect.

## Conclusion and Future Perspectives

Rising antimicrobial resistance and lack of varied treatment modalities have led to a growing interest in developing nanotechnology-based treatment strategies for infectious diseases. Silica nanoparticles, in particular, shows a huge potential for infectious disease treatment due to its versatile and tunable characteristic features. Controllable pore properties of SiNPs have enabled loading of a wide variety of payloads including drugs, dyes and metals leading to both therapeutic and diagnostic application. Tunable surface modifications of SiNPs have enabled both covalent and non-covalent modes of interaction with the payload and thus facilitating a well-controlled release and activity of the functional payloads. SiNPs have also demonstrated a good efficacy against notorious biofilms which warrants its application in antibiofilm coating and wearable implants. However, payload delivery using nanomaterials suffers from issues such as protein fouling, immunogenicity and toxicity. The recent FDA approval of investigation new drug (IND) application of fluorescent core-shell SiNPs called Cornell dots (C-dots) (Kim et al., 2016) for diagnostic purposes in cancer is an encouraging new step endorsing the safety profile of SiNPs.

Overall, the report encompasses the role of SiNPs as an efficient drug delivery vehicle with a great potential in infectious disease treatment. Use of nanotechnology for infectious diseases treatment when compared to cancer therapeutics, is still at an early stage of understanding and development. There is still more room for learning and implementing new notions for achieving better efficacy without trading off its safety and biocompatibility. Dealing with bacterial and biofilm microenvironments can be tricky, and the mode of action differs from species to species. Understanding this can help us design suitable stimuli-responsive drug delivery systems that cater explicitly to any particular disease treatment. One interesting characteristic of SiNPs is its adaptable nature that allows them to blend in with other nanomaterials including iron oxide nanoparticles, polymeric nanoparticles, metal nanoparticles and even with liposomes. More research could be devoted to this area of hybrid silica nanoparticles that can make use of the benefits from both worlds.

## Author Contributions

VS designed and wrote this study. SO and PE reviewed the manuscript. All authors contributed to the article and approved the submitted version.

## Conflict of Interest

The authors declare that the research was conducted in the absence of any commercial or financial relationships that could be construed as a potential conflict of interest.

## Abbreviations

SiNPs: Silica nanoparticles
MSN: Mesoporous silica nanoparticles
AMR: Antimicrobial resistance
CDC: Centers for disease control and prevention
MRSA: Methicillin-resistant *Staphylococcus aureus*
CRE: Carbapenem-resistant *Enterobacteriaceae*
HMSN: Hollow mesoporous silica nanoparticles
TEOS: Tetraethylorthosilicate
APTES: (3-Aminopropyl) triethoxysilane
MPTMS: (3-Mercaptopropyl) trimethoxysilane
CTAB: Cetyltrimethylammoniumbromide
O/W: Oil-in-water
W/O: Water-in-oil
NIR: Near-infrared
C-dots: Cornell dots
FDA: Food and drug administration
IND: Investigational new drug
NLR: Long rod nanoparticles
NSR: Short rod nanoparticles
NS: Spherical nanoparticles
Si-OH: Silanol
PEG: Polyethylene glycol
AMAS: *N*-(α-maleimidoacetoxy) succinimide ester
MBS: *m*-Maleimido-benzoyl-*N*-hydroxysuccinimide ester
Mal-PEG-NHS: Maleimide-PEG-*N*-hydroxysuccinimide
VTES: Vinyltriethoxysilane
FITC: Fluorescein isothiocyanate
SPECT/CT: Single-photon emission computed tomography
LDH: Lactate dehydrogenase
ROS: Reactive oxygen species
NO: Nitric oxide
TB: Tuberculosis
TESPC: 3-(Triethoxysilyl)propyl isocyanate
EDC: 1-Ethyl-3-(3-dimethylaminopropyl) carbodiimide
NHS: *N*-hydroxysuccinimide
PEI: Polyethyleneimine
OCT: Octenidine dihydrochloride
TC: Tetracycline
CHX: Chlorhexidine
MXF: Moxifloxacin
IAAH-Ag: Silver-indole-3-acetic acid hydrazide
MBI: 1-Methyl-1H-benzimidazole
β-CD: β-Cyclodextrin
ANA: Aniloalkane
PCL: Polycaprolactone
WHO: World Health Organization
INH: Isoniazid
RIF: Rifampicin
CFZ: Clofazimine
PZA: Pyrazinamide
AP: Acetophenone
CS-DDS: Composite scaffold drug delivery system
β-TCP: β-Tricalcium phosphate
Cht: Chitosan
AEMP3: (Aminoethylaminomethyl)phenethyltrimethoxysilane
AHAP3: *N*-(6-aminohexyl) aminopropyltrimethoxysilane
AEAP3: *N*-(2-aminoethyl)-3-aminopropyltrimethoxysilane
MAP3: *N*-methylaminopropyltrimethoxysilane
PROLI/NO: 1-(2-(carboxylate) pyrrolidin-1-yl) diazen-1-ium-1,2-diolate
PCN: Phenazine-1-carboxamide
PFTE: Polytetrafluoroethylene
PMMA: Poly(methyl methacrylate).
