# Supplementary material for: Silica Nanoparticles—A Versatile Tool for the Treatment of Bacterial Infections
Source: Front Chem. 2020 Jul 15;8:602. doi: 10.3389/fchem.2020.00602 (PMC7374024; doi:10.3389/fchem.2020.00602)
Supplement: Supplementary file 1 [file Table_1.DOCX]

| Supplementary Material | | | | | | |
| --- | --- | --- | --- | --- | --- | --- |
| **Supplementary Table 1:** Summary of silica nanoparticles used as nanocarriers for infectious disease treatment | | | | | | |
| **No.** | **Type of SiNPs** | **Particle shape, size (S) and pore size (P)** | **Surface modification** | **Payloads** | **Organism tested against** | **Ref.** |
| **Drug delivery** | | | | | | |
| 1. | Non-porous SiNPs | Spherical  S: 130±30 nm | Triclosan-(3-(triethoxysilyl)propyl) carbamate (TTESPC) | Triclosan (Irgasan) | *S. aureus*,  *E. coli* | (Makarovsky et al., 2011) |
| 2 | MSN | Spherical  S: 77±23 nm | Aminopropyl silane (APS) | Vancomycin, FITC | *S. aureus,*  *E. coli* | (Qi, Li, Yu, & Wang, 2013) |
| 3 | Non-porous SiNPs | Spherical  S: 72 ± 10 nm  P: 2–3 nm | FITC-doped and carboxylated using APTES with succinic anhydride | Lysozyme | *E. coli* | (Li & Wang, 2013) |
| 4 | Calcined MSN (MSNc) | Spherical  S: 294.6 ± 20.9 nm  P: 3.1– 3.5 nm | - | LL-37 | *E. coli* | (Braun et al., 2016) |
|  | Non-porous SiNPs (NSN) | Spherical  S: 307.9 ± 39.1 nm | - |  |  |  |
|  | Aminated MSN (MSNa) | Spherical  S: 306.9 ± 17.3 nm  P: 3.1– 3.5 nm | Aminopropyltrimethoxy silane  (APTMS) |  |  |  |
| 5. | Porous silicon (pSiNPs) | S: ̴ 200 nm  P: 14.4 nm | Amine modification with (3-aminopropyl)-dimethyl-ethoxy silane | Lactoferrin-dKK | *P. aeruginosa* | (Kwon et al., 2017) |
|  |  |  | Carboxyl modification with succinic anhydride |  |  |  |
|  |  |  | Phosphonate modification using 3-(trihydroxysilyl)propyl methylphosphonate |  |  |  |
| 6. | MCM-41 | Spherical  S: ̴113 nm  P: 4.1 nm | [3-(2- aminoethyl amino) propyl]trimethoxysilane (EDS)/Succinic anhydride/ polyethylenimine (PEI) | Nisin | *S. aureus* | (Behzadi et al., 2018) |
| 7. | Drug templated-MCM-41 | S: 71±9 nm  P: 1.4, 1.7 and 2.0 nm (Trimodal) | - | Octenidine dihydrochloride (OCT) | *S. mutans* | (Stewart, Finer, & Hatton, 2018) |
| 8. | SiNPs | Spherical  S: 58 nm | - | Tetracycline (TC) | *E. coli* | (Capeletti et al., 2014) |
| 9. | MCM-41 | Spherical  S: 120 nm  P: 2.4 nm | *N*-(2-aminoethyl)-3-aminopropyltriethoxysilane (DAMO) | Levofloxacin (LEVO) | *S. aureus* and its biofilm | (Pedraza, Díez, Isabel-Izquierdo-Barba, Colilla, & Vallet-Regí, 2018) |
| 10. | MCM-41 | Spherical  S: 122–190 nm  P: 2.3 nm | Aminopropyl silanetriol (APST)  Trihydroxy-silyl-propyl-methyl-phospho nate (THSPMP) | levofloxacin | *E. coli*,  *S. aureus* | (Encinas et al., 2019) |
| 11. | IBN-4 | Cylindrical  S: 175 nm  P: 5.95 nm | Triethoxysilyl butyraldehyde | Indole-3 acetic acid hydrazide-Silver coordination complex (IAAH-Ag) | *S. epidermidis,*  *B. subtilis,*  *S. aureus*,  *E. coli* | (Kuthati, Kankala, Lin, Weng, & Lee, 2015) |
| 12. | MSN | Spherical  S: 80nm  P: 3.5 nm | Folic acid (FA) and Calcium phosphate (CaP) | Ampicillin | *E. coli*,  *S. aureus* | (Chen et al., 2018) |
| 13. | MSN with disulfide bridge framework | S: ̴100 nm | Silver-decorated | Chlorhexidine (CHX) | *Streptococcus mutans* and its biofilm | (Lu et al., 2018) |
| 14. | MCM-41 | Spherical  S: 100 nm | α-Cyclodextrin + aniloalkane (ANA) | Moxifloxacin  (MXF) | *F. tularensis* | ( Li et al., 2015) |
|  |  |  | β-Cyclodextrin + 1-methyl-1H-benzimidazole (MBI) |  |  |  |
| 15 | MCM-41 | Spherical  S: 177.5 nm  P: 2.7 nm | 3-(Mercaptopropyl) triethoxysilane and SA20 Aptamer-gated | Vancomycin | *S. aureus,*  *S. epidermidis* | (Kavruk et al., 2015) |
| 16. | SiO_2_-Fe_3_O_4_ NPs | Spherical  P: 6.07 nm | Poly(*N*-isopropylacrylamide) (PNIPAM) | Lysozyme | *B. cereus*  *M. luteus* | (Yu et al., 2015) |
| 17. | MSN | Spherical  S: 500 nm  P: 0.2 nm | - | Malachite green (MG) | *E. coli*,  *S. aureus* | (Paramanantham, Siddhardha, et al., 2019) |
| 18. | MSN | Spherical  S: 500 nm  P: 0.2 nm | - | Toludine ble (TB) | *S. aureus,*  *P. aeruginosa* | (Paramanantham, Antony, et al., 2019) |
| 19. | MSN | Spherical  S: 660 nm  P: 2.95 nm | trichloro (1H, 1H, 2H, 2H- heptadecafluorodecyl) silane (THFS) | Methylene blue (MB) | *E. coli*,  *S. aureus* | (Sun et al., 2020) |
| 20. | MCM-41 | Spherical  S: 119.0±8.5 nm  P: 4.02 nm | *N*-(2-aminoethyl)-3-aminopropyltrimethoxysilane (AAPTMS)/ | Polymyxin B  (PmB) | *E. coli, Acinetobacter baumannii,*  *P. aeruginosa* | (Gounani et al., 2018) |
| 21. | Non-porous SiNPs | Spherical  S: ̴180 nm | Polyallylamine | Polyethelene glycol and vancomycin-conjugated poly (acrylic acid) (PAA-PEG-Van) and Cypate (Cy) | Methicillin-resistant  *S. aureus* | (Zhao et al., 2017) |
| 22. | Silica xerogel | S: 190-395 nm  P: 9.6 nm | PEG | Gentamicin  (GTMC) | *Salmonella serovar Typhimurium* | (Seleem et al., 2009) |
| 23. | Non-porous SiNPs | Spherical  S: 100, 300 & 500 nm | Silica-collagen nanocomposites | Rifamycin and Gentamicin | *S. aureus,*  *P. aeruginosa* | (Alvarez et al., 2014) |
| 24. | Silica and paramagnetic SiNPs | - | Hydrophilic copolymer brushes made of oligo (ethylene glycol) side chains | Magainin peptide | *L. ivanovii* | (Blin et al., 2011) |
| 25. | Calcined MSN (MSNc) | Spherical  S: 294.6±20.9 nm  P: 3.1-3.5 nm | Aminopropyltrimethoxysilane (APTMS) for MSNc and MSNa | LL-37 | *E. coli* | (Braun et al., 2016) |
|  | Aminated nanoparticles (MSNa) | Spherical  S: 307.9±39.1 nm  P: 3.1-3.5 nm |  |  |  |  |
|  | Non-porous SiNPs | Spherical  S: 306.9±17.3 nm |  |  |  |  |
| 26. | MSN | Spherical  S: 150 nm  P: 2.4 nm | 3-(2-aminoethylamino) propyl] trimethoxysilane (DAMO) & G3-PPI dendrimer | Levofloxacin  (LEVO) | *E. coli* and its biofilm | (González et al., 2018) |
| 27. | Non-porous SiNPs | Spherical  S: 327±10 nm | - | Gentamicin  (GTMC) | *B. subtilis,*  *P. fluorescens* and *E. coli* | (Mosselhy et al., 2016) |
| 28. | MSN | - | - | Gentamicin  (GTMC) | *S. aureus* and its biofilm | (Tamanna et al., 2018) |
| 29. | MCM-41 | Spherical  S: 100 nm  P: 2.56 nm, | *n*-[(3-trimethoxysilyl) propyl] ethylendiamine triacetic acid trisodium salt & capped with ε-poly-L-lysine | Rhodamine B or histidine kinase autophosphorylation inhibitors (HKAId | *E. coli,*  *S.marcescens* | (Velikova et al., 2017) |
| **Anti-tuberculosis therapy** | | | | | | |
| 30. | MSN | Spherical  S: ̴ 400 nm  P: 2.65 nm | Porous beta-tricalcium phosphate (β-TCP) scaffold | Isoniazid (INH) and rifampicin (RFP) | *M. tuberculosis* | (Zhu et al., 2011) |
| 31. | MSN | Spherical  S: 100 nm  P: 2 nm | Polyethyleneimine (PEI) and  β-cyclodextrin | Isoniazid (INH) and rifampicin (RIF) | *M. tuberculosis* | (Clemens et al., 2012) |
| 32. | AMS-6 | Spherical  S: 300 nm  P: 5.49 nm | Aminopropyl triethoxysilane (APES) | PA-824 | *M. tuberculosis* | (Xia et al., 2014) |
| 33. | MSN | Spherical  S: 100 nm, 50 nm  P: 2 nm | Poly (ethylene imine)-poly (ethylene glycol) (PEI-PEG) | Isoniazid (INH) | *M. tuberculosis* | (Hwang et al., 2015) |
| 34. | MSN | Spherical  S: 165.7 nm  P: 2.8 nm | Acetophenone (AP) as a chaperone | Clofazimine (CFZ) | *M. tuberculosis* | (Chen et al., 2018) |
| 35. | MSN | Spherical  S: 200 nm  P: 3 nm | APTMS | Peptide NZX | *M. bovis* | (Tenland et al., 2019) |
| **Metal-silica nanocomposites** | | | | | | |
| 36. | MCM-41 | Spherical  S: 100 nm | APTES | Silver nanocrystals | *B. anthracis,*  *E.coli* | (Liong, France, Bradley, & Zink, 2009) |
| 37. | MSP1 (MCM-41) | Spherical  S: ̴ 200 nm  P: 3.8 nm | Electrostatic adsorption of chitosan | Silver ions (Ag+) | *E. coli* | (Karaman et al., 2016) |
|  | MSP2  (SBA-15) | Rod-shaped  S: 475 nm  P: 11.4 nm |  |  |  |  |
|  | MSP4  (SBA-15) | Rod-shaped  S: ̴ 500 nm  P: 14.2 nm |  |  |  |  |
| 38. | MSN | Spherical  S: 61.1 ±7.9 nm  P: 2.6 nm | - | Silver nanoclusters | *S. aureus,*  *P. aeruginosa* | (Liu, Li, Fang, & Zhu, 2019) |
| 39. | MCM-41 | - | - | Silver nanoparticles | *P. aeruginosa, E. coli, C. albicans* | (Abou-El-Sherbini et al., 2018) |
| 40. | SBA-15 | - | Chitosan-SiNPs nanocomposites | Silver nanoparticles | *E. coli*,  *S. aureus* | (Zienkiewicz-Strzałka et al., 2020) |
| 41. | SiNPs | - | - | Copper | *E. coli*,  *B. subtilis* | (Maniprasad & Santra, 2012) |
| 42. | SiNPs | Spherical  S: 88.48±9.85 nm | - | Copper | *S. aureus*  *E. ccloacae*  *E. coli*  *P.citrinum* | (Kim et al., 2006) |
| 43. | MSN | Spherical  S: 89 ± 3 nm  P: 4.64 | Triethoxysilylpropylmaleamic acid | Copper | *S. aureus*  *E. coli* | (Díaz-García et al., 2019) |
| 44. | SBA-15 | Rod-shaped  S: length-1.3 μm width-500 nm | - Triethoxysilylpropylmaleamic acid - Triethoxy-3-(2-imidazolin-1-yl)propylsilane | Copper | *S. aureus*  *E. coli* | (Díaz-García et al., 2020) |
| 45. | SiNPs | Spherical  P: 212 nm | O-carboxymethyl chitosan-Schiff’s base (OCMC*HN) | Copper | *E. coli,*  *B. subtilis* | (Naz et al., 2018) |
| 46. | SiNPs | Spherical  S: 200±10nm | - | Copper | *E. coli*  *S. aureus* | (Zhang et al., 2010) |
| 47. | MSN | Bean-like morphology  S: length-1.7, width-0.9 µm | APTES (Amino group)  Succinic anhydride (Carboxyl group) | Gold | *E. coli*  *S. aureus* | (Tao, Ju, Ren, & Qu, 2015) |
| 48. | MCM-41 | Spherical  P: 1.72 nm | - | Titanium (Ti), Vanadium (V), Palladium (Pd) | *E. coli,*  *B. subtilis,*  *S. aureus,*  *S. paratyphi* | (Chirra et al., 2019) |
| 49. | MSN | Spherical  S: <100 nm | APTMS | Copper (II)  Nickel (II) | *B. subtilis,*  *S. aureus,*  *E. coli,*  *P. aeruginosa* | (Tahmasbi, Sedaghat, Motamedi, & Kooti, 2018) |
| 50. | SiO_2_-Fe_3_O_4_ NPs | Spherical  S: ∼32 nm | Polyvinylalcohol (PVA) & hexapeptide (Gly-Ala-Phe-Pro-His-Arg) | Vancomycin | *S. aureus,*  *E. coli* | (Zhang et al., 2020) |
| 51. | SiNPs | Spherical  S: 20−25 nm | 4-cyanopenta-noic acid dithiobenzoate (CPDB) | Cobaltocenium (CoAEMAPF_6_) metallopolymer | *S. aureus,*  *E. coli,*  *P. aeruginosa,*  *K. pneumonia,*  *P. vulgaris* | (Pageni et al., 2018) |
| **Nitric oxide (NO) delivery** | | | | | | |
| 52. | Non-porous  SiNPs | S: 136±15 nm | *N*-diazeniumdiolated *N*-(6- aminohexyl) aminopropyltrimethoxysilane (AHAP3) | Nitric oxide (NO) | *P. aeruginosa* | (Hetrick et al., 2008) |
| 53. | Non-porous  SiNPs | S:136 ± 15 nm, 90 ± 10 nm | *N*-(6-aminohexyl)aminopropyl- trimethoxysilane (AHAP3)  *N*-Methylaminopropyltrimethoxysilane (MAP3) | Nitric oxide (NO) | *P. aeruginosa,*  *E. coli,*  *S. aureus,*  *S. epidermidis,*  *C. albicans* | (Hetrick, Shin, Paul, & Schoenfisch, 2009) |
| 54. | Non-porous  SiNPs | S: 50 nm, 100 nm, 200 nm | *N*-diazeniumdiolated-*N*-(6 aminohexyl) aminopropyltrimethoxysilane  (AHAP3) | Nitric oxide (NO) | *P. aeruginosa* | (Carpenter et al., 2011) |
| 55. | Non-porous  SiNPs | Spherical  S: 180±26 nm | Quaternary ammonium epoxides (QA-epoxides) with *N*-diazeniumdiolated-*N*-(6 aminohexyl) aminopropyltrimethoxysilane  (AHAP3/NO) | Nitric oxide (NO) | *S. aureus* and  *P. aeruginosa* | (Carpenter et al., 2012) |
| 56. | Non-porous  SiNPs | Spherical & Rod-like  S: 14 nm, 50 nm, 150 nm | *N*-diazeniumdiolated-*N*-(6 aminohexyl) aminopropyltrimethoxysilane  (AHAP3) and N-(2-aminoethyl)-3-amino-isobutyl- dimethyl-methoxysilane (AEAI) | Nitric oxide (NO) | Biofilms of *S. aureus* and  *P. aeruginosa* | (Slomberg et al., 2013) |
| **Antibiofilm coatings/dental composites** | | | | | | |
| 57. | MSNP | Spherical  S: 193 nm  P: 6.43 nm | Silicone urethral catheters coated with PCN-MSNPs | Phenazine-1-carboxamide (PCN) | *S. aureus,*  *C. albicans* and its biofilms | (Kanugala, Jinka, Puvvada, Banerjee, & Kumar, 2019) |
| 58. | Silica-gentamicin (SG) nanohybrid | Spherical  S: 298.1±12.2 nm, | Cross-linked gelatin/SG composite coating on percutaneous titanium implants | Gentamicin  (GTMC) | *S. aureus* and its biofilm | (Wang et al., 2017) |
| 59 | SiO_2_ | Spherical  S: 125±18.12 nm | APTES  Divinyl sulfone (DVS) | Polyethylenimine (PEI) | *L. monocytogenes* biofilm | (Huang, Chen, Nugen, & Goddard, 2016) |
| 60. | MSN | Spherical | Poly-L-glutamic acid (PG), Polyallylamine hydrochloride (PAH) were | Silver | *S. aureus* | (Ding et al., 2020) |
| 61. | MSN | Rod-like  S: 100 nm  P: 6.2 nm | Polymethylmethacrylate (PMMA) bone cement matrix | Gentamicin (GTMC) | *S. aureus* | (Slane et al., 2014) |
| 62. | MSN | Rod-shaped  S: 100-600 nm length and 100 nm in diameter  P: 6.2 nm | Polymethylmethacrylate (PMMA) bone cement matrix | Gentamicin  (GTMC) | *S. aureus* | (Letchmanan et al., 2017) |
| 63. | MSN | Spherical  S: 85.2 ± 7.7 nm  P: 3.54 ± 0.41 nm | Polymethylmethacrylate (PMMA) bone cement matrix | Amphotericin B  (AmB) | *C. albicans,*  *S. oralis* | (Lee et al., 2016) |
| 64. | MSN | Spherical  S: 85.2 ± 7.7 nm  P: 3.54 ± 0.41 nm | Polymethylmethacrylate (PMMA) bone cement matrix | Silver-sulfadiazine (AgSD) | *C. albicans,*  *S. oralis* | (Jo et al., 2017) |
| 65. | MCM-41 | P: 2.5 nm | Coated with polydimethylsiloxane and layered on PMMA matrix | Chlorhexidine (CHX) | *-* | (Mai et al., 2019) |
| 66. | MSN | Spherical  S: 200 nm  P: 4 nm | Poly(e-caprolactone) (PCL) electrospun fibres | Levofloxacin  (LEVO) | *E. coli* | (Jalvandi et al., 2015) |

**References**

Abou-El-Sherbini, K. S., Amer, M. H. A., Abdel-Aziz, M. S., Hamzawy, E. M. A., Sharmoukh, W., & Elnagar, M. M. (2018). Encapsulation of Biosynthesized Nanosilver in Silica Composites for Sustainable Antimicrobial Functionality. *Global Challenges*, *2*(10), 1800048. https://doi.org/10.1002/gch2.201800048

Alvarez, G. S., Hélary, C., Mebert, A. M., Wang, X., Coradin, T., & Desimone, M. F. (2014). Antibiotic-loaded silica nanoparticle-collagen composite hydrogels with prolonged antimicrobial activity for wound infection prevention. *Journal of Materials Chemistry B*, *2*(29), 4660–4670. https://doi.org/10.1039/c4tb00327f

Behzadi, F., Darouie, S., Alavi, S. M., Shariati, P., Singh, G., Dolatshahi-Pirouz, A., & Arpanaei, A. (2018). Stability and antimicrobial activity of nisin-loaded mesoporous silica nanoparticles: a game-changer in the war against maleficent microbes. *Journal of Agricultural and Food Chemistry*, *66*(16), 4233–4243. https://doi.org/10.1021/acs.jafc.7b05492

Blin, T., Purohit, V., Leprince, J., Jouenne, T., & Glinel, K. (2011). Bactericidal microparticles decorated by an antimicrobial peptide for the easy disinfection of sensitive aqueous solutions. *Biomacromolecules*, *12*(4), 1259–1264. https://doi.org/10.1021/bm101547d

Braun, K., Pochert, A., Lindén, M., Davoudi, M., Schmidtchen, A., Nordström, R., & Malmsten, M. (2016). Membrane interactions of mesoporous silica nanoparticles as carriers of antimicrobial peptides. *Journal of Colloid and Interface Science*, *475*, 161–170. https://doi.org/10.1016/j.jcis.2016.05.002

Capeletti, L. B., De Oliveira, L. F., Gonçalves, K. D. A., De Oliveira, J. F. A., Saito, Â., Kobarg, J., … Cardoso, M. B. (2014). Tailored silica-antibiotic nanoparticles: Overcoming bacterial resistance with low cytotoxicity. *Langmuir*, *30*(25), 7456–7464. https://doi.org/10.1021/la4046435

Carpenter, A. W., Slomberg, D. L., Rao, K. S., & Schoenfisch, M. H. (2011). Influence of scaffold size on bactericidal activity of nitric oxide-releasing silica nanoparticles. *ACS Nano*, *5*(9), 7235–7244. https://doi.org/10.1021/nn202054f

Carpenter, A. W., Worley, B. V, Slomberg, D. L., & Schoenfisch, M. H. (2012). Dual action antimicrobials: Nitric oxide release from quaternary ammonium-functionalized silica nanoparticles. *Biomacromolecules*, *13*(10), 3334–3342. https://doi.org/10.1021/bm301108x

Chen, W., Cheng, C. A., Lee, B. Y., Clemens, D. L., Huang, W. Y., Horwitz, M. A., & Zink, J. I. (2018). Facile strategy enabling both high loading and high release amounts of the water-insoluble drug clofazimine using mesoporous silica nanoparticles. *ACS Applied Materials and Interfaces*, *10*(38), 31870–31881. https://doi.org/10.1021/acsami.8b09069

Chen, X., Liu, Y., Lin, A., Huang, N., Long, L., Gang, Y., & Liu, J. (2018). Folic acid-modified mesoporous silica nanoparticles with pH-responsiveness loaded with Amp for an enhanced effect against anti-drug-resistant bacteria by overcoming efflux pump systems. *Biomaterials Science*, *6*(7), 1923–1935. https://doi.org/10.1039/c8bm00262b

Chirra, S., Siliveri, S., Gangalla, R., Goskula, S., Gujjula, S. R., Adepu, A. K., … Narayanan, V. (2019). Synthesis of new multivalent metal ion functionalized mesoporous silica and studies of their enhanced antimicrobial and cytotoxicity activities. *Journal of Materials Chemistry B*, *7*(45), 7235–7245. https://doi.org/10.1039/c9tb01736d

Clemens, D. L., Lee, B.-Y., Xue, M., Thomas, C. R., Meng, H., Ferris, D., … Horwitz, M. A. (2012). Targeted intracellular delivery of antituberculosis drugs to Mycobacterium tuberculosis-infected macrophages via functionalized mesoporous silica nanoparticles. *Antimicrobial Agents and Chemotherapy*, *56*(5), 2535–2545. https://doi.org/10.1128/aac.06049-11

Díaz-García, D., Ardiles, P. R., Díaz-Sánchez, M., Mena-Palomo, I., del Hierro, I., Prashar, S., … Gómez-Ruiz, S. (2020). Copper-functionalized nanostructured silica-based systems: Study of the antimicrobial applications and ROS generation against gram positive and gram negative bacteria. *Journal of Inorganic Biochemistry*, *203*. https://doi.org/10.1016/j.jinorgbio.2019.110912

Díaz-García, D., Ardiles, P. R., Prashar, S., Rodríguez-Diéguez, A., Páez, P. L., & Gómez-Ruiz, S. (2019). Preparation and study of the antibacterial applications and oxidative stress induction of copper maleamate-functionalized mesoporous silica nanoparticles. *Pharmaceutics*, *11*(1), 30. https://doi.org/10.3390/pharmaceutics11010030

Ding, Y., Hao, Y., Yuan, Z., Tao, B., Chen, M., Lin, C., … Cai, K. (2020). A dual-functional implant with an enzyme-responsive effect for bacterial infection therapy and tissue regeneration. *Biomaterials Science*, *8*(7), 1840–1854. https://doi.org/10.1039/c9bm01924c

Encinas, N., Angulo, M., Astorga, C., Colilla, M., Izquierdo-Barba, I., & Vallet-Regí, M. (2019). Mixed-charge pseudo-zwitterionic mesoporous silica nanoparticles with low-fouling and reduced cell uptake properties. *Acta Biomaterialia*, *84*, 317–327. https://doi.org/10.1016/j.actbio.2018.12.012

González, B., Colilla, M., Díez, J., Pedraza, D., Guembe, M., Izquierdo-Barba, I., & Vallet-Regí, M. (2018). Mesoporous silica nanoparticles decorated with polycationic dendrimers for infection treatment. *Acta Biomaterialia*, *68*, 261–271. https://doi.org/10.1016/j.actbio.2017.12.041

Gounani, Z., Asadollahi, M. A., Meyer, R. L., & Arpanaei, A. (2018). Loading of polymyxin B onto anionic mesoporous silica nanoparticles retains antibacterial activity and enhances biocompatibility. *International Journal of Pharmaceutics*, *537*(1–2), 148–161. https://doi.org/10.1016/j.ijpharm.2017.12.039

Hetrick, E. M., Shin, J. H., Paul, H. S., & Schoenfisch, M. H. (2009). Anti-biofilm efficacy of nitric oxide-releasing silica nanoparticles. *Biomaterials*, *30*(14), 2782–2789. https://doi.org/10.1016/j.biomaterials.2009.01.052

Hetrick, E. M., Shin, J. H., Stasko, N. A., Johnson, C. B., Wespe, D. A., Holmuhamedov, E., & Schoenfisch, M. H. (2008). Bactericidal efficacy of nitric oxide-releasing silica nanoparticles. *ACS Nano*, *2*(2), 235–246. https://doi.org/10.1021/nn700191f

Huang, K., Chen, J., Nugen, S. R., & Goddard, J. M. (2016). Hybrid antifouling and antimicrobial coatings prepared by electroless co-deposition of fluoropolymer and cationic silica nanoparticles on stainless steel: efficacy against Listeria monocytogenes. *ACS Applied Materials and Interfaces*, *8*(25), 15926–15936. https://doi.org/10.1021/acsami.6b04187

Hwang, A. A., Lu, J., Tamanoi, F., & Zink, J. I. (2015). Functional nanovalves on protein-coated nanoparticles for in vitro and in vivo controlled drug delivery. *Small*, *11*(3), 319–328. https://doi.org/10.1002/smll.201400765

Jalvandi, J., White, M., Truong, Y. B., Gao, Y., Padhye, R., & Kyratzis, I. L. (2015). Release and antimicrobial activity of levofloxacin from composite mats of poly(ɛ-caprolactone) and mesoporous silica nanoparticles fabricated by core–shell electrospinning. *Journal of Materials Science*, *50*(24), 7967–7974. https://doi.org/10.1007/s10853-015-9361-x

Jo, J. K., El-Fiqi, A., Lee, J. H., Kim, D. A., Kim, H. W., & Lee, H. H. (2017). Rechargeable microbial anti-adhesive polymethyl methacrylate incorporating silver sulfadiazine-loaded mesoporous silica nanocarriers. *Dental Materials*, *33*(10), e361–e372. https://doi.org/10.1016/j.dental.2017.07.009

Kanugala, S., Jinka, S., Puvvada, N., Banerjee, R., & Kumar, C. G. (2019). Phenazine-1-carboxamide functionalized mesoporous silica nanoparticles as antimicrobial coatings on silicone urethral catheters. *Scientific Reports*, *9*(1). https://doi.org/10.1038/s41598-019-42722-9

Karaman, D. S., Sarwar, S., Desai, D., Björk, E. M., Odén, M., Chakrabarti, P., … Chakraborti, S. (2016). Shape engineering boosts antibacterial activity of chitosan coated mesoporous silica nanoparticle doped with silver: A mechanistic investigation. *Journal of Materials Chemistry B*, *4*(19), 3292–3304. https://doi.org/10.1039/c5tb02526e

Kavruk, M., Celikbicak, O., Ozalp, V. C., Borsa, B. A., Hernandez, F. J., Bayramoglu, G., … Arica, M. Y. (2015). Antibiotic loaded nanocapsules functionalized with aptamer gates for targeted destruction of pathogens. *Chem. Commun.*, *51*(40), 8492–8495. https://doi.org/10.1039/C5CC01869B

Kim, Y. H., Lee, D. K., Cha, H. G., Kim, C. W., Kang, Y. C., & Kang, Y. S. (2006). Preparation and characterization of the antibacterial Cu nanoparticle formed on the surface of SiO2 nanoparticles. *Journal of Physical Chemistry B*, *110*(49), 24923–24928. https://doi.org/10.1021/jp0656779

Kuthati, Y., Kankala, R. K., Lin, S. X., Weng, C. F., & Lee, C. H. (2015). pH-triggered controllable release of silver-indole-3 mesoporous Silica nanoparticles (IBN-4) for effectively killing malignant bacteria. *Molecular Pharmaceutics*, *12*(7), 2289–2304. https://doi.org/10.1021/mp500836w

Kwon, E. J., Skalak, M., Bertucci, A., Braun, G., Ricci, F., Ruoslahti, E., … Bhatia, S. N. (2017). Porous silicon nanoparticle delivery of tandem peptide anti-infectives for the treatment of Pseudomonas aeruginosa lung infections. *Advanced Materials*, *29*(35), 1–9. https://doi.org/10.1002/adma.201701527

Lee, J. H., El-Fiqi, A., Jo, J. K., Kim, D. A., Kim, S. C., Jun, S. K., … Lee, H. H. (2016). Development of long-term antimicrobial poly(methyl methacrylate) by incorporating mesoporous silica nanocarriers. *Dental Materials*, *32*(12), 1564–1574. https://doi.org/10.1016/j.dental.2016.09.001

Letchmanan, K., Shen, S.-C. C., Ng, W. K., Kingshuk, P., Shi, Z., Wang, W., & Tan, R. B. H. (2017). Mechanical properties and antibiotic release characteristics of poly(methyl methacrylate)-based bone cement formulated with mesoporous silica nanoparticles. *Journal of the Mechanical Behavior of Biomedical Materials*, *72*, 163–170. https://doi.org/10.1016/j.jmbbm.2017.05.003

Li, L., & Wang, H. (2013). Enzyme-coated mesoporous silica nanoparticles as efficient antibacterial agents in vivo. *Advanced Healthcare Materials*, *2*(10), 1351–1360. https://doi.org/10.1002/adhm.201300051

Li, Z., Clemens, D. L., Lee, B. Y., Dillon, B. J., Horwitz, M. A., & Zink, J. I. (2015). Mesoporous Silica Nanoparticles with pH-Sensitive Nanovalves for Delivery of Moxifloxacin Provide Improved Treatment of Lethal Pneumonic Tularemia. *ACS Nano*, *9*(11), 10778–10789. https://doi.org/10.1021/acsnano.5b04306

Liong, M., France, B., Bradley, K. A., & Zink, J. I. (2009). Antimicrobial activity of silver nanocrystals encapsulated in mesoporous silica nanoparticles. *Advanced Materials*, *21*(17), 1684–1689. https://doi.org/10.1002/adma.200802646

Liu, J., Li, S., Fang, Y., & Zhu, Z. (2019). Boosting antibacterial activity with mesoporous silica nanoparticles supported silver nanoclusters. *Journal of Colloid and Interface Science*, *555*, 470–479. https://doi.org/10.1016/j.jcis.2019.08.009

Lu, M., Ge, Y., Qiu, J., Shao, D., Zhang, Y., Bai, J., … Tang, C. (2018). Redox/pH dual-controlled release of chlorhexidine and silver ions from biodegradable mesoporous silica nanoparticles against oral biofilms. *International Journal of Nanomedicine*, *Volume 13*, 7697–7709. https://doi.org/10.2147/ijn.s181168

Mai, Kim, Hyun, Park, Lee, & Lee. (2019). A New Antibacterial Agent-Releasing Polydimethylsiloxane Coating for Polymethyl Methacrylate Dental Restorations. *Journal of Clinical Medicine*, *8*(11), 1831. https://doi.org/10.3390/jcm8111831

Makarovsky, I., Boguslavsky, Y., Alesker, M., Lellouche, J., Banin, E., & Lellouche, J. P. (2011). Novel triclosan-bound hybrid-silica nanoparticles and their enhanced antimicrobial properties. *Advanced Functional Materials*, *21*(22), 4295–4304. https://doi.org/10.1002/adfm.201101557

Maniprasad, P., & Santra, S. (2012). Novel copper (Cu) loaded core-shell silica nanoparticles with improved Cu bioavailability: synthesis, characterization and study of antibacterial properties. *Journal of Biomedical Nanotechnology*, *8*(4), 558–566. Retrieved from http://www.ncbi.nlm.nih.gov/pubmed/22852465

Mosselhy, D. A., Ge, Y., Gasik, M., Nordström, K., Natri, O., & Hannula, S. P. (2016). Silica-gentamicin nanohybrids: Synthesis and antimicrobial action. *Materials*, *9*(3), 1–16. https://doi.org/10.3390/ma9030170

Naz, A., Arun, S., Narvi, S. S., Alam, M. S., Singh, A., Bhartiya, P., & Dutta, P. K. (2018). Cu(II)-carboxymethyl chitosan-silane schiff base complex grafted on nano silica: Structural evolution, antibacterial performance and dye degradation ability. *International Journal of Biological Macromolecules*, *110*, 215–226. https://doi.org/10.1016/j.ijbiomac.2017.11.112

Pageni, P., Yang, P., Chen, Y. P., Huang, Y., Bam, M., Zhu, T., … Tang, C. (2018). Charged Metallopolymer-Grafted Silica Nanoparticles for Antimicrobial Applications. *Biomacromolecules*, *19*(2), 417–425. https://doi.org/10.1021/acs.biomac.7b01510

Paramanantham, P., Antony, A. P., Sruthil Lal, S. B., Sharan, A., Siddhardha, B., Kasinathan, K., … Syed, A. (2019). Antimicrobial photodynamic activity of toluidine blue encapsulated in mesoporous silica nanoparticles against Pseudomonas aeruginosa and Staphylococcus aureus. *Biofouling*, *35*(1), 89–103. https://doi.org/10.1080/08927014.2019.1570501

Paramanantham, P., Siddhardha, B., Lal, S. B. S., Sharan, A., Alyousef, A. A., Al Dosary, M. S., … Syed, A. (2019). Antimicrobial photodynamic therapy on Staphylococcus aureus and Escherichia coli using malachite green encapsulated mesoporous silica nanoparticles: An in vitro study. *PeerJ*, *2019*(9), e7454. https://doi.org/10.7717/peerj.7454

Pedraza, D., Díez, J., Isabel-Izquierdo-Barba, Colilla, M., & Vallet-Regí, M. (2018). Amine-functionalized mesoporous silica nanoparticles: A new nanoantibiotic for bone infection treatment. *Biomedical Glasses*, *4*(1), 1–12. https://doi.org/10.1515/bglass-2018-0001

Qi, G., Li, L., Yu, F., & Wang, H. (2013). Vancomycin-modified mesoporous silica nanoparticles for selective recognition and killing of pathogenic Gram-positive bacteria over macrophage-like cells. *ACS Applied Materials and Interfaces*, *5*(21), 10874–10881. https://doi.org/10.1021/am403940d

Seleem, M. N., Munusamy, P., Ranjan, A., Alqublan, H., Pickrell, G., & Sriranganathan, N. (2009). Silica-antibiotic hybrid nanoparticles for targeting intracellular pathogens. *Antimicrobial Agents and Chemotherapy*, *53*(10), 4270–4274. https://doi.org/10.1128/AAC.00815-09

Slane, J., Vivanco, J., Meyer, J., Ploeg, H. L., & Squire, M. (2014). Modification of acrylic bone cement with mesoporous silica nanoparticles: Effects on mechanical, fatigue and absorption properties. *Journal of the Mechanical Behavior of Biomedical Materials*, *29*, 451–461. https://doi.org/10.1016/j.jmbbm.2013.10.008

Slomberg, D. L., Lu, Y., Broadnax, A. D., Hunter, R. A., Carpenter, A. W., & Schoenfisch, M. H. (2013). Role of size and shape on biofilm eradication for nitric oxide-releasing silica nanoparticles. *ACS Applied Materials and Interfaces*, *5*(19), 9322–9329. https://doi.org/10.1021/am402618w

Stewart, C. A., Finer, Y., & Hatton, B. D. (2018). Drug self-assembly for synthesis of highly-loaded antimicrobial drug-silica particles. *Scientific Reports*, *8*(1), 1–12. https://doi.org/10.1038/s41598-018-19166-8

Sun, J., Fan, Y., Zhang, P., Zhang, X., Zhou, Q., Zhao, J., & Ren, L. (2020). Self-enriched mesoporous silica nanoparticle composite membrane with remarkable photodynamic antimicrobial performances. *Journal of Colloid and Interface Science*, *559*, 197–205. https://doi.org/10.1016/j.jcis.2019.10.021

Tahmasbi, L., Sedaghat, T., Motamedi, H., & Kooti, M. (2018). Mesoporous silica nanoparticles supported copper(II) and nickel(II) Schiff base complexes: Synthesis, characterization, antibacterial activity and enzyme immobilization. *Journal of Solid State Chemistry*, *258*, 517–525. https://doi.org/10.1016/j.jssc.2017.11.015

Tamanna, T., Landersdorfer, C. B., Ng, H. J., Bulitta, J. B., Wood, P., & Yu, A. (2018). Prolonged and continuous antibacterial and anti-biofilm activities of thin films embedded with gentamicin-loaded mesoporous silica nanoparticles. *Applied Nanoscience (Switzerland)*, *8*(6), 1471–1482. https://doi.org/10.1007/s13204-018-0807-8

Tao, Y., Ju, E., Ren, J., & Qu, X. (2015). Bifunctionalized mesoporous silica-supported gold nanoparticles: Intrinsic oxidase and peroxidase catalytic activities for antibacterial applications. *Advanced Materials*, *27*(6), 1097–1104. https://doi.org/10.1002/adma.201405105

Tenland, E., Pochert, A., Krishnan, N., Rao, K. U., Kalsum, S., Braun, K., … Godalyid, G. (2019). Effective delivery of the anti-mycobacterial peptide NZX in mesoporous silica nanoparticles. *PLoS ONE*, 1–16. https://doi.org/10.1371/journal.pone.0212858

Velikova, N., Mas, N., Miguel-Romero, L., Polo, L., Stolte, E., Zaccaria, E., … Wells, J. (2017). Broadening the antibacterial spectrum of histidine kinase autophosphorylation inhibitors via the use of ε-poly-L-lysine capped mesoporous silica-based nanoparticles. *Nanomedicine: Nanotechnology, Biology, and Medicine*, *13*(2), 569–581. https://doi.org/10.1016/j.nano.2016.09.011

Wang, J., Wu, G., Liu, X., Sun, G., Li, D., & Wei, H. (2017). A decomposable silica-based antibacterial coating for percutaneous titanium implant. *International Journal of Nanomedicine*, *12*, 371–379. https://doi.org/10.2147/IJN.S123622

Xia, X., Pethe, K., Kim, R., Ballell, L., Barros, D., Cechetto, J., … Garcia-Bennett, A. (2014). Encapsulation of anti-tuberculosis drugs within mesoporous silica and intracellular antibacterial activities. *Nanomaterials*, *4*(3), 813–826. https://doi.org/10.3390/nano4030813

Yu, E., Galiana, I., Martínez-Máñez, R., Stroeve, P., Marcos, M. D., Aznar, E., … Amorós, P. (2015). Poly(N-isopropylacrylamide)-gated Fe3O4/SiO2 core shell nanoparticles with expanded mesoporous structures for the temperature triggered release of lysozyme. *Colloids and Surfaces B: Biointerfaces*, *135*, 652–660. https://doi.org/10.1016/j.colsurfb.2015.06.048

Zhang, N., Gao, Y., Zhang, H., Feng, X., Cai, H., & Liu, Y. (2010). Preparation and characterization of core-shell structure of SiO2@Cu antibacterial agent. *Colloids and Surfaces B: Biointerfaces*, *81*(2), 537–543. https://doi.org/10.1016/j.colsurfb.2010.07.054

Zhang, W., Taheri-Ledari, R., Hajizadeh, Z., Zolfaghari, E., Ahghari, M. R., Maleki, A., … Tian, Y. (2020). Enhanced activity of vancomycin by encapsulation in hybrid magnetic nanoparticles conjugated to a cell-penetrating peptide. *Nanoscale*, *12*(6), 3855–3870. https://doi.org/10.1039/c9nr09687f

Zhao, Z., Yan, R., Yi, X., Li, J., Rao, J., Guo, Z., … Chen, C. (2017). Bacteria-activated theranostic nanoprobes against methicillin-resistant Staphylococcus aureus infection. *ACS Nano*, *11*(5), 4428–4438. https://doi.org/10.1021/acsnano.7b00041

Zhu, M., Wang, H., Liu, J., He, H., Hua, X., He, Q., … Shi, J. (2011). A mesoporous silica nanoparticulate/β-TCP/BG composite drug delivery system for osteoarticular tuberculosis therapy. *Biomaterials*, *32*(7), 1986–1995. https://doi.org/10.1016/j.biomaterials.2010.11.025

Zienkiewicz-Strzałka, M., Deryło-Marczewska, A., Skorik, Y. A., Petrova, V. A., Choma, A., & Komaniecka, I. (2020). Silver nanoparticles on chitosan/silica nanofibers: Characterization and antibacterial activity. *International Journal of Molecular Sciences*, *21*(1), 166. https://doi.org/10.3390/ijms21010166
